# The rise of genomics in snake venom research: recent advances and future perspectives

**DOI:** 10.1093/gigascience/giac024

**Published:** 2022-04-01

**Authors:** Wei-qiao Rao, Konstantinos Kalogeropoulos, Morten E Allentoft, Shyam Gopalakrishnan, Wei-ning Zhao, Christopher T Workman, Cecilie Knudsen, Belén Jiménez-Mena, Lorenzo Seneci, Mahsa Mousavi-Derazmahalleh, Timothy P Jenkins, Esperanza Rivera-de-Torre, Si-qi Liu, Andreas H Laustsen

**Affiliations:** Department of Biotechnology and Biomedicine, Technical University of Denmark, Søltofts Plads 224, 2800 Kongens Lyngby, Denmark; Department of Mass Spectrometry, Beijing Genomics Institute-Research, 518083, Shenzhen, China; Department of Biotechnology and Biomedicine, Technical University of Denmark, Søltofts Plads 224, 2800 Kongens Lyngby, Denmark; Trace and Environmental DNA (TrEnD) Laboratory, School of Molecular and Life Sciences, Curtin University, Kent Street, 6102, Bentley Perth, Australia; Globe Institute, University of Copenhagen, Øster Voldgade 5, 1350, Copenhagen, Denmark; Globe Institute, University of Copenhagen, Øster Voldgade 5, 1350, Copenhagen, Denmark; Department of Mass Spectrometry, Beijing Genomics Institute-Research, 518083, Shenzhen, China; Department of Biotechnology and Biomedicine, Technical University of Denmark, Søltofts Plads 224, 2800 Kongens Lyngby, Denmark; Department of Biotechnology and Biomedicine, Technical University of Denmark, Søltofts Plads 224, 2800 Kongens Lyngby, Denmark; DTU Aqua, Technical University of Denmark, Vejlsøvej 39, 8600, Silkeborg, Denmark; Department of Biotechnology and Biomedicine, Technical University of Denmark, Søltofts Plads 224, 2800 Kongens Lyngby, Denmark; Trace and Environmental DNA (TrEnD) Laboratory, School of Molecular and Life Sciences, Curtin University, Kent Street, 6102, Bentley Perth, Australia; Department of Biotechnology and Biomedicine, Technical University of Denmark, Søltofts Plads 224, 2800 Kongens Lyngby, Denmark; Department of Biotechnology and Biomedicine, Technical University of Denmark, Søltofts Plads 224, 2800 Kongens Lyngby, Denmark; Department of Mass Spectrometry, Beijing Genomics Institute-Research, 518083, Shenzhen, China; Department of Biotechnology and Biomedicine, Technical University of Denmark, Søltofts Plads 224, 2800 Kongens Lyngby, Denmark

**Keywords:** snake genomics, DNA sequencing, venom, venom evolution, snakes, snake toxins

## Abstract

Snake venoms represent a danger to human health, but also a gold mine of bioactive proteins that can be harnessed for drug discovery purposes. The evolution of snakes and their venom has been studied for decades, particularly via traditional morphological and basic genetic methods alongside venom proteomics. However, while the field of genomics has matured rapidly over the past 2 decades, owing to the development of next-generation sequencing technologies, snake genomics remains in its infancy. Here, we provide an overview of the state of the art in snake genomics and discuss its potential implications for studying venom evolution and toxinology. On the basis of current knowledge, gene duplication and positive selection are key mechanisms in the neofunctionalization of snake venom proteins. This makes snake venoms important evolutionary drivers that explain the remarkable venom diversification and adaptive variation observed in these reptiles. Gene duplication and neofunctionalization have also generated a large number of repeat sequences in snake genomes that pose a significant challenge to DNA sequencing, resulting in the need for substantial computational resources and longer sequencing read length for high-quality genome assembly. Fortunately, owing to constantly improving sequencing technologies and computational tools, we are now able to explore the molecular mechanisms of snake venom evolution in unprecedented detail. Such novel insights have the potential to affect the design and development of antivenoms and possibly other drugs, as well as provide new fundamental knowledge on snake biology and evolution.

## Background

**Figure 1:**
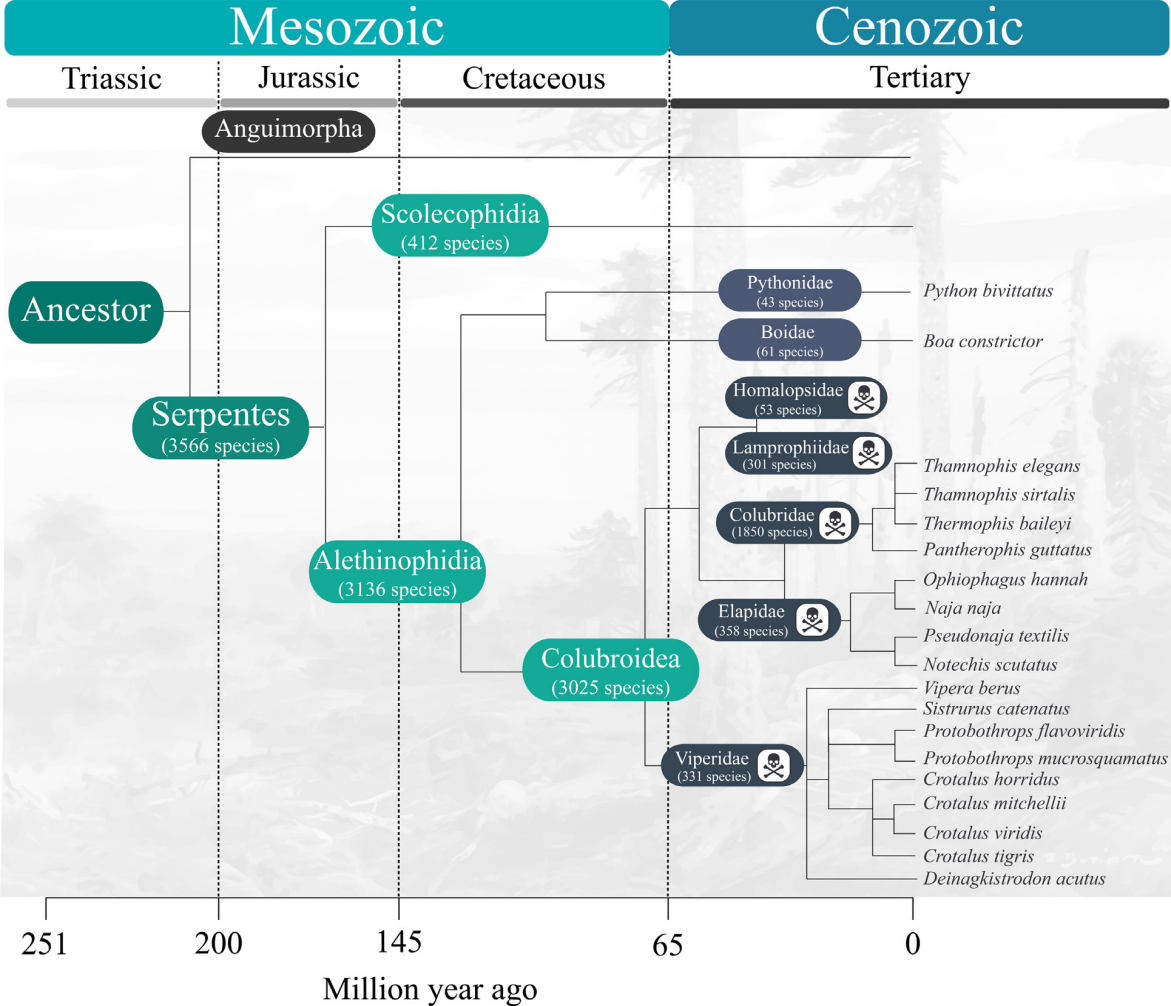
Schematic diagram of snake evolution based on data from Reptile-database.org [16]. Snakes (Serpentes) are divided into 3 main infraorders, Scolecophidia, Henophidia, and Alethinophidia, which together encompass ∼24 families (7 shown here). Families comprising venomous species are indicated with a skull and crossbones symbol. Colubridae constitutes the largest family of snakes, encompassing 52% of the ∼3,566 snake species currently described. The total number of currently described venomous snake species is 2,901, predominantly falling within the families Homalopsidae, Lamprophiidae, Colubridae, Elapidae, and Viperidae. Only snake species that have undergone whole-genome sequencing and assembly are listed in this figure.

Snakes (Squamata: Serpentes) represent a monophyletic lineage, comprising ∼3,600 extant species found in all continents, except Antarctica [1,2]. From an evolutionary perspective, these reptiles stand out for their characteristic lack of limbs, elongated body shape, and exclusively carnivorous diet. Even before the advent of genetic approaches, conventional anatomical and morphology-based phylogenetic evidence unambiguously suggested that snakes are nested within lizards, with the Anguimorpha lineage (e.g., monitor lizards, glass lizards, beaded lizards) as their closest relatives [3–5]. Together with amphisbaenians, snakes and all other lizards thus form the largest branch of terrestrial vertebrates, the squamate reptiles [3–5]. Snakes have many specialized adaptations compared to other reptile lineages. For example, the evolution of infrared sensing pits in pit vipers (Viperidae: Crotalinae), boas (Boidae), and pythons (Pythonidae), and of a venom apparatus in several snake families (Fig. 1), provides these animals with exceptional predatory capabilities despite the loss of limbs and the degradation of visual and auditory perception in many (but not all) species [6–8]. Moreover, severe jaw modifications and low metabolic rates enable snakes to swallow and digest large prey whole, further consolidating their position as formidable predators [9,10]. Thus, snakes are important model organisms for evolutionary studies and have yielded insights into limb development [11–13], sex chromosome evolution [14], and venom evolution [15].

The development of next-generation sequencing (NGS) technologies in recent decades has allowed researchers to generate large genomic datasets and rendered the assembly and characterization of complete genomes a routine task. Despite the availability of NGS since the early 2000s, the use of these technologies to assemble and study complete snake genomes has been limited, especially when compared to the amount of research that has been conducted in the fields of snake venom proteomics and transcriptomics [17]. It was not until 2013 that the first snake genomes based on high-throughput sequencing data were published for the Burmese python (*Python bivittatus*), the red-tailed boa (*Boa constrictor constrictor*), and the king cobra (*Ophiophagus hannah*) [9, 18,19]. Fortunately, snake genome research has eventually gained more attention, with 18 new genomes being released since 2013 and several more currently in progress [15,20–31]. These increased sequencing efforts have already revealed intriguing insights into the regulation and expression of venom-related genes. As an example, a large number of dormant toxin-encoding genes with unknown bioactivity were identified in the Okinawan habu (*Protobothrops flavoviridis*) [15]. Such discoveries could be of high scientific value and may improve our basic understanding of the interplay between protein function and evolution. Furthermore, because toxins from several animal lineages are known to possess different types of bioactivity, some of them could find utility in a variety of applications, from the development of novel therapeutics [32] to biopesticides [33] and molecular research tools [34]. With only 21 snake genomes publicly available to date, there is great unexplored scientific potential in sequencing and analysing more snake genomes [17,35].

From a broader perspective, having access to a complete or nearly complete assembled genome provides an excellent basis for addressing a wide range of biological research questions. For example, genomic data can be used to predict protein-coding exons [36] (including exons in genes that recently underwent pseudogenization), non-expressed genes, translated proteins, and microRNA genes [37]. Genomic data may also allow for the identification of toxin orthologs using comparative studies and homology searches [38]. Knowledge of homology is crucial for the reliability of functional annotation of genomes and can provide fundamental information on evolution and speciation processes [39,40]. Therefore, complete genomes are crucial to the field of proteomics as well, because the absence of reliable genome-derived protein libraries forces researchers to rely on homologous proteins from other organisms as a benchmark against which to compare newly characterized protein sequences. This results in severely limited accuracy in identifying potentially homologous proteins, which consequently leads to overlooking and/or misrepresenting evolutionary patterns. This is especially relevant considering the likely widespread occurrence of alternative splicing in snake genomes, which gives rise to multiple messenger RNA products that in turn result in various isoforms of a particular toxin [41–43]. Extensive post-genomic and post-translational modifications are also at play, leading to often remarkable discrepancies between genome, transcriptome, and proteome in terms of expression and sequence identity [44–46]. Along this line, comparative analysis of whole snake genomes could likely provide invaluable insight on the evolution and structure of the gene regulatory network responsible for the expression of venom genes in these animals (and arguably venomous amniotes in general) [47].

Several approaches are available to obtain reliable genomic data. Among them is reduced-representation sequencing, in which only a part of the genome is sequenced [48]. For instance, capture sequencing techniques allow for specific areas of interest (e.g., the exon part of the genome) to be targeted and sequenced at a lower cost compared to whole-genome sequencing (WGS) [49]. Although less suitable for studying venom genes, restriction site associated–DNA sequencing (RAD-seq) uses restriction enzymes to obtain genome-wide sequencing data, which have recently been used to study population demographic trends that underly venom variation [50]. Nevertheless, reliable detection of homologous genes across species and/or lineages can be hindered by the acquisition, loss, or pseudogenization of genes [40]. One way to overcome this challenge is to use WGS, which represents a more comprehensive resource for the detection of homologous genes because it provides the entire genotype of the target organism(s) [40]. WGS can also provide information on genomic variability of a species, and potentially discover and quantify the extent of selective (e.g., positive/purifying selection and hitchhiking effects) and neutral forces (e.g., genetic drift) driving venom evolution [51].

This review aims to provide a comprehensive summary of the current knowledge on snake genomics, with a particular focus on the current use and future potential of high-throughput DNA sequencing technologies in the field of snake toxinology. Moreover, we discuss how these technologies can be used to expand our current knowledge on snake venom evolution and toxin diversification.

## Current Status of Snake Venom Research

### Overview of snake toxin families

Studies have estimated that between 19,000 and 25,000 toxins are found in venoms from the Elapidae and Viperidae snake families, but only a few thousands have been characterized [52]. Nonetheless, this body of knowledge has proven sufficient for the systematic classification of snake venom toxins into 63 families, most of which are, however, only found in a small percentage of snake species and/or in negligible amounts within venom mixtures [53]. The 4 families generally considered to be of highest relevance both from a clinical (human envenoming cases) and an ecological perspective (e.g., prey incapacitation) are the 3-finger toxins (3FTxs), phospholipases A_2_ (PLA_2_s), snake venom metalloproteinases (SVMPs), and snake venom serine proteinases (SVSPs). Other widespread snake venom protein families include cysteine-rich secretory proteins (CRISPs), L-amino acid oxidases (LAAOs), and C-type lectin-like proteins (CTLPs) [53]. An overview of the main snake venom toxin families is provided in Table 1.

**Table 1:** Number of toxin-encoding genes for 22 toxin families in selected venomous and non-venomous reptile species

Venom protein family	Non-venomous				Venomous							
	Venom family abbreviation	Anolis carolinensis*	*Boa constrictor*	*Python bivittatus*	*Thamnophis sirtalis*	*Ophiophagus hannah*	*Naja naja*	*Deinagkistrodon acutus*	*Protobothrops flavoviridis*	*Crotalus viridis*	*Crotalus tigris*	*Bothrops jararaca*
5′-nucleotidases	5Nase	1	1	1		1	2	1	1	5		1
Acetylcholinesterase	ACeH	22	11	12		16	2	14		7		
Kunitz-type peptide		86	39	49		53	3	70		2	2	
Bradykinin-potentiating peptides and C-type natriuretic peptides	BNP	1	3	1		6	3	2	1	1	1	1
Cysteine-rich secretory proteins	CRISPs	2	1	1	2	3	7	2	2	4	2	1
C-type lectins and C-type lectin-like proteins	CTLPs	5	7	6		13	2	22	10	6	5	6
Disintegrins	Dis							3	2			
Factor V		5	5	6		5		5		3		
Factor X		9	11	11		11		11				
Hyaluronidases	HYAL	5	6	6	1	6	3	6	1	4	1	1
L-amino acid oxidases	LAAO	4	5	6	2	3	3	4	1	4	2	2
Nerve growth factors or neurotrophins	NGF	5	5	5		5	3	4	1	2	1	1
Phosphodiesterases	PDE	6	6	5		5	1	5	1		1	
Phospholipases A_2_	PLA_2_	1	1	1	1	4	8	1	9	5	3	1
Phospholipases B	PLB	1	1	1		4	1	1	1		1	1
Snake venom metalloproteinases	SVMP (PI)		2					1				
	SVMP (PII)		1					4	3		3	7
	SVMP (PIII)	1	1	2	7	4	8	5	6	11	2	20
Snake venom serine proteinases	SVSP	4	6	7	1	8	8	22	11	9	15	12
Three-finger toxins	3FTx					5	19		4	2	3	
Vascular endothelial growth factors	VEGF	4	7	7		5	6	6	1	3	1	1
Venom ficolins	Veficolins	11	9	9		11		10		4	1	
Vespryns/ohanin-like proteins		90	40	52		39	1	42	1		1	
Waprin		5	3	3		4		3		1	1	

In venomous snake genomes, the numbers refer to the venom gland genome only. Non-venomous species lack venom glands, and the indicated numbers refer to homologous proteins expressed in other organs.

* The green anole (*Anolis carolinensis*) was selected as outgroup taxon because it is a non-venomous, non-snake squamate with a complete genome sequence available.

3FTxs belong to a superfamily of non-enzymatic proteins and are a major component in the venoms of most elapids, while they generally feature less prominently in viperid and colubrid venoms. These toxins have 3 β-stranded loops extending from a central core, contain 4 or 5 conserved disulfide bonds, and cause a wide range of pharmacological effects [54–56]. A prominent group of 3FTxs, α-neurotoxins, interfere with neuromuscular signal transmission of cholinergic neurons by binding to nicotinic acetylcholine receptors, causing flaccid paralysis [53, 55]. Other 3FTxs are toxic to cardiomyocytes and can lead to increased heart rate and ultimately cardiac arrest, while yet others function as calcium channel blockers or platelet aggregation inhibitors [54].

PLA_2_s are found in the venoms of vipers, elapids, and certain rear-fanged species [57–60] and exert a wide variety of cytotoxic, myotoxic, cardiotoxic, and neurotoxic effects [57,58,60]. Of particular interest is a catalytically inactive, myotoxic category of PLA_2_s stemming from a single substitution of a highly conserved amino acid residue (Asp49 to Lys49/Asn49) [57]. Both non-catalytic and enzymatic PLA_2_s are able to form heterodimeric complexes with other PLA_2_s or other toxins in certain venoms, whereby their toxicity is greatly potentiated [58]. Most snake genomes contain multiple PLA_2_ genes, which likely originated from repeated gene duplication events [60,61]. These paralogs have diverse pharmacological activities, which were likely acquired through neofunctionalization (i.e., recruitment of a paralog to the venom gland following gene duplication and its subsequent evolution into a toxin-coding gene) [62,63]. Pseudogenization and deletion of PLA_2_ genes are also frequent in snakes, making this toxin family one of the most dynamic in terms of evolutionary history [28,39,64]. The annotation of more snake genomes, and the likely consequent discovery of more PLA_2_ genes, might provide an improved understanding of the evolution and the mechanisms of action of these proteins (including how the phenomenon of toxin synergism has evolved) and potentially assist in the characterization of similar evolutionary processes for other enzymes.

Another major category of enzymes found in snake venoms are SVMPs [65,66]. These proteinases are enzymes that cleave peptide bonds in other proteins, which may result in the degradation or activation of the target [66]. Zinc-dependent SVMPs are often the major venom component in vipers [67], and these toxins hydrolyze extracellular matrix components, leading to rupture of capillaries and local and systemic bleeding [59]. Other clinical manifestations induced by SVMPs include edema, inflammation, myonecrosis, and reduced muscle regeneration [67]. Additionally, these enzymatic toxins can have anticoagulant, clotting factor–activating, or platelet-aggregating effects [68,69]. SVMPs are divided into 3 distinct classes depending on the domains present in the mature enzymes: P-I (metalloproteinase [M] domain only), P-II (M domain and disintegrin-like domain), and P-III (M domain, disintegrin-like domain, and cysteine-rich domain) [65]. Elucidation of snake genomes could help shed light on how these enzymes evolved from the ancestral P-III type via loss of domains [70–72] and postgenomic modifications, acquiring different functions and specificities in the process [65]. A better understanding of SVMP evolution via snake genomics could also provide insight into the evolutionary process that led to the diversification of SVMPs as a whole from the ancestral A disintegrin and metalloprotease (ADAM) family of metalloproteinases, which play significant roles in all stages of development and survival of higher-order organisms [73].

Finally, SVSPs are typically present in the venoms of vipers [74] but can also be found in elapid venoms [75]. SVSPs contain 2 6-stranded β-barrels and consist of ∼245 amino acid residues. SVSPs also have a unique extended C-terminus that forms a disulfide bridge, which contributes to structural stability [76]. These toxins can induce blood coagulation through fibrin formation, Factor V activation, prothrombin activation, actin dissolvement, or platelet aggregation; conversely, they can also act as anticoagulants via fibrinolysis, fibrinolytic enzyme activation, or protein C activation [59, 77–79]. This toxin family has received increased attention with recent genome studies on *P. flavoviridis* and *B. jararaca*, where the evolutionary pathway as well as the molecular regulation of SVSP expression was systematically investigated [15, 29].

In summary, snake toxin families are numerous and their pharmacological actions are complex [80]. Knowledge on the toxicity and structure of different snake toxin families is essential to further our understanding of snake venom evolution, as well as to understand venoms as drug targets for antivenom development. Much knowledge has been gained from venom proteomics and transcriptomics, and new genomics technologies now allow for the investigation of the evolutionary relationships between toxins in different families in unprecedented detail.

### State of the art in snake genomics

With the rapid development of high-throughput sequencing technology, large-scale genomic projects have generated rich sequence information data of billions of base pairs and have paved the way for a new era in the field of phylogenetics, whereby the evolutionary history of organisms can be reconstructed from genomic data. The supermatrix method is the most well-known approach for analysing concatenation of multiple gene sequences, and using genomic data sets with improved resolution can potentially mitigate phylogenetic problems previously caused by sampling errors [81]. However, because only 21 (∼0.6%) of the ∼3,600 existing snake species have undergone WGS so far [9,15, 17,18,20–28,30,82–85], snake genomics will likely develop significantly in the coming years. A complete list of currently available snake genomes is provided in Table 2.

**Table 2:** Whole-genome sequencing studies on snakes, published or in progress

						Assembly				Annotation	Venomous	INSDC ID	Source
Scientific classification				Sequencing�		Scaffold							
Superfamily	Family	Genus	Species	Sequencing platform	DoC	GC%	N50 Size (kb)	Contig N50 Size (kb)	Genome Size (Gb)	Protein-encoding genes identified			
Colubroidea	Viperidae	*Bothrops*	*jararaca*	Illumina; PacBio; BAC-SeqSc	150 IL20 PB			163.5	2.1		Yes	PRJNA691605	[29]
		*Crotalus*	*viridis*	Illumina; PacBio	100	36.6	139	15.74	1.3		Yes	PDHV00000000.1	[20,21]
			*tigris*	Illumina; PacBio	190 IL33 PB	39.9/39.8	207,720	2,110	1.6	18,240	Yes	VORL00000000	[28]
			*pyrrhus*	Illumina	40	38.5	5.1	4.1	1.1		Yes	JPMF00000000.1	[26]
			*horridus*	Illumina	135	34.3	23.8	5.8	1.5		Yes	LVCR00000000.1	[82]
		*Protobothrops*	*flavoviridis*	Illumina	96	38.2	467	3.8	1.4	20,540	Yes	BFFQ00000000.1	[15]
			*mucrosquamatus*	Illumina	86	40.6	424	22	1.6	20,122	Yes	BCNE00000000.2	[24]
		*Sistrurus*	*catenatus*	Illumina, PacBio			1,045,000		1.6		Yes	PRJNA750087	[31]
		*Vipera*	*berus*	Illumina	121	41.3	126.6	11.7	1.5		Yes	JTGP00000000.1	[25]
		*Deinagkistrodon*	*acutus*	Illumina	♂ 114 ♀ 238		2,120	22.42	1.4	21,194	Yes	DQ343647.1	[86]
	Colubridae	*Pantherophis*	*guttatus*	Illumina	13	38.3	4.3	2.39	1.4		No	JTLQ00000000.1	[22]
		*Thermophis*	*baileyi*	Illumina	185	43.6	2,414	16.8	1.8	20,995	No	QLTV00000000	[23]
		*Thamnophis*	*sirtalis*	Illumina	72	41.8	516	10.45	1.4		Yes	LFLD00000000.1	[87]
			*elegans*	Illumina; PacBio	62	41.1	100,851	4,620	1.6	18,900	Yes	PRJNA561996	
	Elapidae	*Ophiophagus*	*hannah*	Illumina	28	40.6	226	3.98	1.6		Yes	AZIM00000000.1	[18]
		*Pseudonaja*	*textilis*		73	40.1	14,685	50.44	1.6	19,358	Yes	ULFR00000000.1	[84]
		*Notechis*	*scutatus*	Illumina; PacBio	71	40.2	5,997	31.76	1.6	19,770	Yes	PRJEB27871	[28]
		*Naja*	*naja*	PacBio; Nanopore; Illumina	250	40.4	223,350	303.98	1.79	23,248	Yes	SOZL00000000.1	[27]
		*Hydrophis*	*curtus*	Illumina NovaSeq	120	37.2	1,346	183	1.62	21,863	Yes	PRJNA597425	[30]
Pythonoidea	Pythonidae	*Python*	*bivittatus*	Illumina; Roche 454	20	39.7	214	10.66	1.4	19,793	No	AEQU00000000.2	[9]
Booidea	Boidae	*Boa*	*constrictor*	Illumina; Roche 454; PacBio	125				1.6		No		[19]

PB stands for PacBio and IL for Illumina.

GC% refers to the percentage of the guanine (G) and cytosine (C) bases in a genome, scaffold N50 is a measure of the assembly quality (see below), DoC is a measure of the depth of coverage (see below), and INSDC ID is the NCBI gene bank accession number of the respective genome.

**Figure 2:**
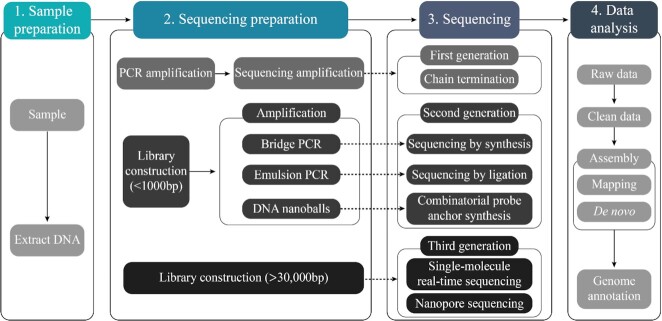
Schematic representation of the next-generation sequencing pipeline for genomic assembly. **(1)** Multiple companies have marketed sequencing platforms for genomic and transcriptomic studies, the most commonly used being Illumina (left), PacBio (middle), and Nanopore (right). **(2)** The 3 platforms differ in read length and accuracy of their generated sequences. Whilst Illumina sequencing generally yields short reads with low error rates, Nanopore sequences are substantially longer (≤2 Mb), yet subject to frequent sequencing errors. Last, PacBio generates sequences with lengths and error rates in between the 2 other platforms. **(3)** After sequencing, reads are computationally processed and assembled into contigs, which in turn **(4)** serve as the building blocks for scaffolds.The scaffolds are then aligned and annotated to produce the complete target genome.

Available snake genomes differ notably in their assembly and annotation qualities, which makes evaluation of genome quality an important factor in determining the suitability of a genome for addressing a given set of questions. For instance, while estimation of nucleotide composition and genomic repeat content can be achieved from a relatively fragmented genome assembly, high-quality genome assemblies are required for analyses of multi-gene families and regulatory elements [88]. The reason for this is that the majority of the known venom gene families form tandem-arrayed “gene islands” (significantly enriched in microchromosomes, see, e.g., [13]), which generally represent a challenge for performing a continuous assembly. To achieve the best quality of assembly of venom genes, the use of long-read technology (e.g., PacBio HiFi or MinIon) is therefore essential (Fig 2). Genome assembly quality is assessed using statistics that measure fragmentation of the genome assembly, such as total assembly length, total contig number, contig N50, and scaffold N50. The total length of the assembly represents the total length of all the contigs that are part of the *de novo* assembled genome. A high total assembly length usually indicates a high-quality genome assembly. The contig N50 expresses the contiguity of the assembled genome. For instance, a contig N50 of 10 kb implies that 50% of the entire genome assembly is contained in contigs that are longer than 10 kb. Thus, a high contig N50 value represents a high-quality assembly without too many gaps. Currently, the contig N50 values of most published snake genomes are <25 kb; exceptions include 7 species with better assembly quality, namely, *T. elegans* (western terrestrial garter snake; 4,620 kb) [89], *C. tigris* (tiger rattlesnake; 2,110 kb) [32], *N. naja* (Indian cobra; 304 kb) [31], *H. curtus* (Shaw's sea snake; 183 kb) [30], *B. jararaca* (Brazilian lancehead; 163.5 kb) [29], *P. textilis* (eastern brown snake; 51 kb) [84], and *N. scutatus* (tiger snake; 32 kb) [83].

Another important parameter is the contig L50, which represents the minimum number of contigs required to cover 50% of the total assembly length. N50 and L50 values can be computed both at the contig and scaffold level. The most complete published snake genomes to date are those of *N. naja* and *C. tigris*, which were assembled by combining data obtained from long-read platforms (Pacific Biosciences [PacBio] and Nanopore) and short-read platforms (Illumina), as well as Chicago, Hi-C, and optical mapping in the case of *N. naja* [31, Fig. 2]. The resulting assemblies have a scaffold N50 reaching a staggering 207.72 Mb (*C. tigris*) and 223.35 Mb (*N. naja*) in length, which is ∼2.5 times greater than the previously assembled human reference genome (87 Mb) [31, 32, 90].

In addition to measures of genome contiguity — such as N50 scores — evaluating the representation of genes in a genome assembly via tools such as BUSCO provides great insight into genome assembly and annotation completeness [91]. A recent study using 611 published eukaryotic genomes showed that assemblies with high contig and scaffold N50 scores were shown to have high BUSCO values as well. However, the study revealed that assemblies with poor N50 scores may also (albeit rarely) show high BUSCO scores [92]. One example of this scenario in snakes is the case of the *P. flavoviridis* genome assembly, where contig N50 was 3.8 kb but percentages of complete and partial coverages for a set of 233 core vertebrate genes were 92.7% and 97.0%, respectively [18].

Furthermore, much can be learned about the quality of a genome from its reported depth of coverage (DoC). A DoC of 10× implies that each position in the genome has been read on average 10 times from independent sequencing reads. High DoC values imply that each position (i.e., each nucleotide) can be determined with greater confidence. Consequently, the 21 snake genomes published to date or in progress can be categorized into 2 groups: (1) a high DoC group (>50×) comprising *B. jararaca* (Brazilian lancehead) [29], *C. viridis* (prairie rattlesnake) [20,21]*, C. horridus* (timber rattlesnake) [82], *P. flavoviridis* [15], *P. mucrosquamatus* (brown-spotted pit viper) [24], *Vipera berus* (European adder) [25], *T. baileyi* (Tibetan hot-spring snake) [23], *T. sirtalis* (common garter snake) [85], *T. elegans* (western terrestrial garter snake), *P. textilis* [84], *C. tigris* (tiger rattlesnake) [28], *N. scutatus* (tiger snake) [93], *D. acutus* (5-paced viper) [86], *B. constrictor* (red-tailed boa) [19]*, H. curtus* (Shaw's sea snake) [30], and *N. naja* [27]; and (2) a low DoC group (13–40×), which includes *S. catenatus* (eastern massasauga rattlesnake), *C. pyrrhus* (southwestern speckled rattlesnake) [26], *P. guttatus* (corn snake) [22], *O. hannah* [18], and *P. bivittatus* [9]. Unsurprisingly, the earliest published snake genomes are characterized by lower DoCs, whereas the more recently sequenced genomes benefitted from technological advancement and thus generally obtained better coverages. The best example of this is the *N. naja* genome, which reached a DoC of 250× [27]—by far the highest DoC reported for a snake genome to date (Table 2). This high DoC enabled the discovery of 43 new toxin-encoding genes, some of which are likely to be unique to *N. naja* [27].

Genome size (the total amount of DNA contained within 1 copy of a single complete genome [94]), number of genes, and guanine-cytosine content [95] vary from species to species and therefore may help elucidate phylogenetic relationships and molecular events (e.g., gene/genome duplication, pseudogenization, gene loss) in the evolution of species. Genome size can vary greatly and is typically correlated with organism size and complexity, as well as with genome repeat content [94]. The reported genome sizes of snakes range from 1.3 to 1.8 Gb, except for *C. pyrrhus* (1.1 Gb) and *B. jararaca* (2.1 Gb) (Table 2). This is consistent with previous findings that squamate reptiles and birds generally have smaller genomes than mammals (1–3 Gb for squamates vs 1–2 Gb for birds vs 2–6 Gb for mammals) (Table 3) [22].

**Table 3:** Selected genomic features compared across several vertebrate lineages [21]

			Transposable elements content (%)	
Tetrapod taxon	Genome size (Gb)	GC content (%)	Range	Mean
Mammals	2.2–6.0	∼40.9	33.4–56.4	44.5
Birds	1.2–2.1	∼40.2	4.6–10.1	7.8
Colubroidea	1.5–3.0	39.3–47.8	33.0–56.3	46.2
Non-colubroid snakes	1.7–2.1	38.8–43.4	28.7–48.7	38.7
Scincoidea (skinks)	1.3–2.6	43.2–46.1	34.3–44.0	37.6

Somewhat counterintuitively, genome size is not necessarily correlated with the number of genes in the genome. For example, although the *Homo sapiens* genome (2.90 Gb) is ∼2 times larger than the *T. sirtalis* genome (1.42 Gb), the number of genes is similar between the two (20,186 genes for *T. sirtalis* compared to 21,407 genes for *H. sapiens*) [87]. This implies higher average gene density (genes/Mb) in *T. sirtalis* than in *H. sapiens*, which is likely rooted in the larger percentage of repeat elements in the human genome compared to that of *T. sirtalis* (∼70% and 37.12%, respectively) [87, 96, 97]. Thus, a considerably larger portion of the *H. sapiens* genome is not composed of protein-coding regions compared to the genome of *T. sirtalis*, which may compensate for the difference between their genome sizes. Furthermore, even though the average gene length of *T. sirtalis* (13,384 bp) is significantly smaller than that of *H. sapiens* (23,247 bp), exon length is comparable between the two (280.12 vs 249.22 bp, respectively) [87].

Unlike their genome sizes and gene lengths, the genomic GC contents for mammals, birds, and squamates are similar (∼40%) (Table 3), and the GC contents of reported snake genomes range from 34.3% to 43.6% (Table 3). Interspecies variation in GC content is thought to be caused by selective variation, mutation bias, and biased DNA repair-related recombination [95]. High GC content might also be an indication of biased sequencing results [98]. It is advisable to obtain information regarding both genome size and GC content prior to *de novo* assembly of a genome because these key genomic features can guide the choice of the most appropriate assembly strategy.

## Understanding Snake Venom Evolution Through Snake Genomes

### Genetic research on snake toxins

Phylogenetics is the cornerstone of our understanding of evolutionary relationships at all taxonomic levels and provides a historical basis for testing and inferring ecological and evolutionary processes [99–102]. In the past few decades, snake venom and its evolutionary origins have received considerable attention [46,103–105]. Although there is uncertainty and controversy about the origin of the venom system in squamate reptiles [29,103,106–108] a prevalent hypothesis is that the core snake venom system evolved in the common ancestor of snakes and lizards [103].

Venom is a polygenic trait that has evolved many times in the tree of life, and it serves a role in both prey capture and defence against predators [104,109]. Unlike many polygenic traits [110,111], venom has a relatively direct pathway from transcription of toxin genes to translation into toxin proteins, which are then stored in the venom gland [46,112]. Thus, by combining venom-gland transcriptomics and venom proteomics, we can accurately map the progression from genotype to phenotype in this adaptive trait [104]. Because transcriptome data will vary depending on the geographical origin of the population, the age and sex of the snake, and time since the last expulsion of venom [113,114] that the snake was subjected to at the time of collection and/or sampling, as well as on the characteristics of the underlying genotype, transcriptomes represent a sample of the spatiotemporally expressed genome and can be used as an entry into genome divergence analysis. Genome divergence analysis takes advantage of whole-genome and/or transcriptome data to reconstruct phylogenies that chart the relationships among snakes, thus representing a precious resource for studies of snake venom evolution.

### Structural characteristics of the toxin genes in snake genomes

More than 10,000 species of squamate reptiles have evolved over the past 200 million years, making this clade a major component of the vertebrate lineage [115]. The number of protein-coding genes is remarkably constant across vertebrates (including snakes), but vertebrate genomes differ considerably in size, structure, and composition [21]. An important genomic feature in this regard are transposable elements (TEs), which are self-replicating DNA sequences with the ability to insert themselves in new positions in the genome, thereby altering genome structure and gene regulation [116,117]. Having a high abundance of TEs could lead to a high degree of evolvability in structural features of the genome, where pseudogenization and gene duplication may occur more frequently, thus creating opportunities for neofunctionalization. As such, it is perhaps hardly surprising that TEs are consistently involved in the evolution of snake venoms [17,18].

Preliminary research indicates that one of the main differences across snake genomes is the abundance and diversity of TEs, which ranges between 33.0–56.3% in Colubroidea and 28.7–8.7% in non-colubroid snakes [20,27,86, 87]. For comparison, other reptiles, such as members of the order Scincoidea, have a lower variation in their number of TEs (34.3–44.0%) (Table 3) [21,27,86]. Both abundance and diversity of TEs in snake genomes are exemplified by the genomes of *D. acutus* and *B. jararaca*. The former is made up of 13.84% long interspersed elements (LINEs, e.g., CR1, L1, and L2), 7.96% DNA transposons (e.g., hAT and TcMar elements), and 2.59% retrotransposons (e.g., Gypsy and DIRS elements) [86], whereas the latter comprises 14.6% LINEs with L2/CR1/Rex as the most abundant (8.8% of whole genome). The observed differences in the repeat content cannot be attributed only to varying sequencing technologies, as shown by the comparison of genome assembly qualities between snakes. For instance, while *B. constrictor* has a higher scaffold N50 (4,505.2 kb) and less total gap length (55,688.38 kb) compared to *D. acutus* (N50 2,122.2 kb; gap length 82,553.36 kb), the latter shows a higher total TE content (47.47 vs 39.59%) [118]. The genomes of *D. acutus* and *O. hannah* have a fairly low divergence level (<10%) of CR1 and hAT elements from the inferred ancestral consensus sequences, while snakes belonging to more basal-branching clades (e.g., *B. constrictor* and *P. bivittatus*) have >20% divergence level [86]. Conversely, CR1 and hAT content is >3 times higher in *D. acutus* and *O. hannah* than in *B. constrictor* and *P. bivittatus*, but the latter 2 species have undergone independent expansion of L2 repeat contents [86]. Another study that highlights genomic differences in TE content in snakes showed that repeat element abundances in the genomes of *D. acutus, T. sirtalis*, and *O. hannah* (all part of the Colubroidea clade) were characterized by a higher CR1-like and DNA transposon content compared to the genome of *P. bivittatus* [87]. Overall, repeat elements in the genomes of venomous snakes are generally more active, diverse, and dynamic compared to those of non-venomous species, indicating that different types of TEs may have played multiple important roles in functional regulation of snake genes throughout evolution.

Another TE category that has attracted research attention is microsatellites (short repeated DNA sequences). Microsatellites are so ubiquitous in certain snake species that a snake genome holds the record for containing the highest microsatellite content in any known eukaryote [21]. Bolstering this claim, a study of 11 viper species found an unprecedented average microsatellite content of 16,214 bp/Mb [21]. In comparison, the mean microsatellite density of 4 non-venomous snakes was ∼55% of that amount, i.e., 8,953 bp/Mb [21]. The same study found that the mean genome density of simple sequence repeat (SSR) loci (448–896 loci/Mb) was roughly twice as large in venomous snake microsatellites as in non-venomous snake homologs [21]. The study further found that the AATAG loci (which tend to be immediately adjacent to CR1-L3 LINEs in colubroid genomes) in venomous colubroids were increased 75-fold compared to other squamate reptiles and 71-fold compared to non-colubroid snakes [21]. Based on the significant expression of SSRs and LINE-SSR hybrid element content in venomous snakes compared to non-venomous snakes, the study also concluded that SSRs and LINE-SSR hybrid elements may have played key roles in the evolution of snake venoms [21]. The dynamics and extent of the influence of SSRs and LINE-SSR on venom evolution therefore represent an intriguing venue for further research.

**Figure 3:**
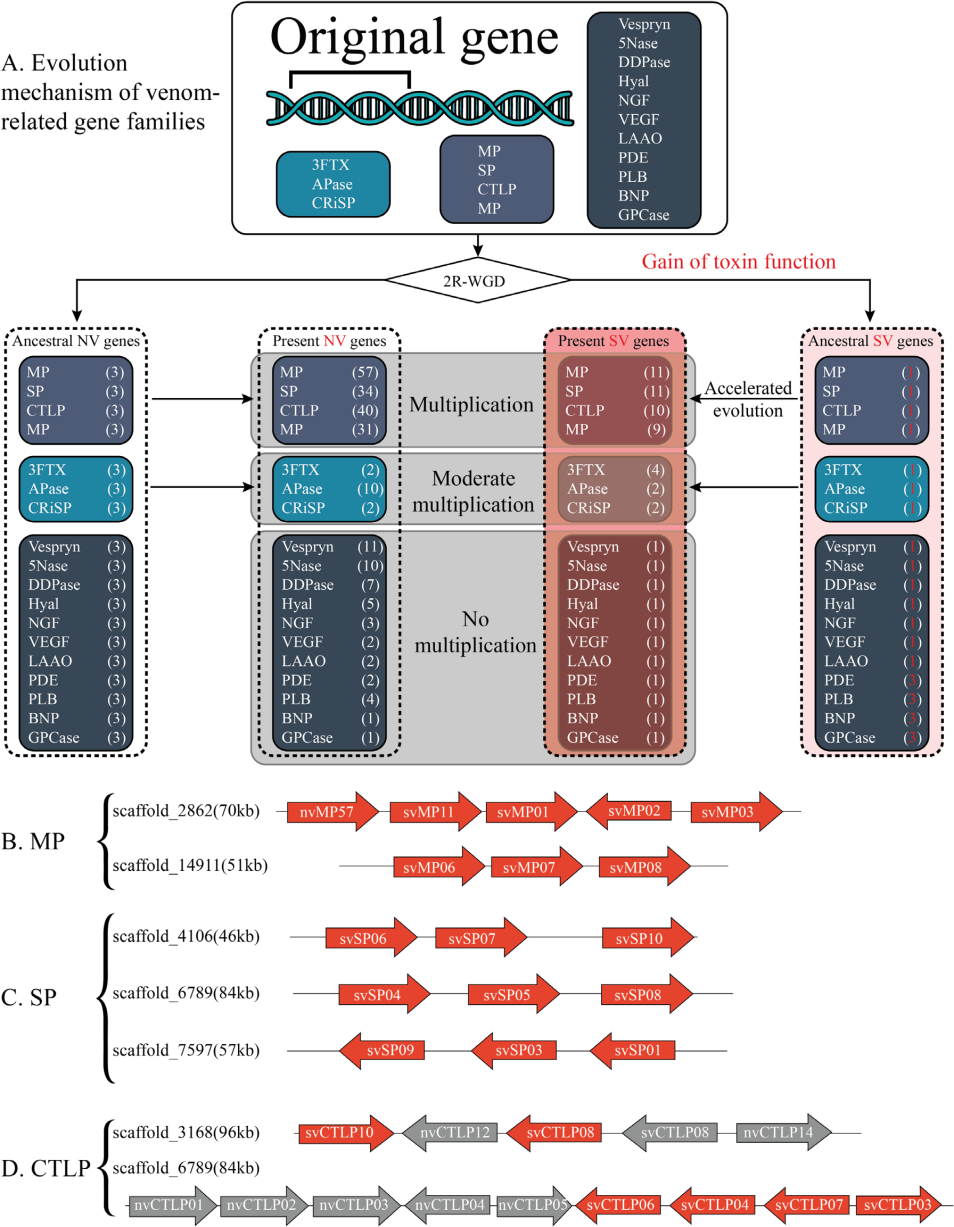
Venom-related gene families in the *P. flavoviridis* genome. **(A)** Deduced evolutionary history of venom-related gene families through 2 rounds of whole-genome duplication (2R-WGD). An original set of 18 genes (shown in the top box) became 72 (4 copies each). Then, a single copy of each family was likely co-opted to develop toxic functions, resulting in 1 snake venom (SV) copy (shown in a pale red box in the right column) and 3 non-venom (NV) paralogs (shown in the see-through box to the left). **(B)** Tandem duplications of SVMP genes. **(C)** Tandem duplications of SVSP genes. **(D)** Tandem duplications of CTLP genes. Based on Fig. 2 and Fig. S8 from [15].

However, microsatellite content alone cannot explain the course of venom evolution. Indeed, another important factor is the chromosomal location of venom genes. What is known about snake chromosomes is largely based on cytogenetic experimental studies, which have revealed that the majority of snakes have 18 chromosomes (8 macrochromosomes and 10 microchromosomes) [20]. It has been observed that a high proportion of venom genes are located on microchromosomes [15,21], revealing a consistent pattern of homologous chromosomal location for multiple venom gene families arranged in tandem-array gene clusters. For example, 37% of all venom genes in the *C. viridis* genome and ∼57% (27/47 genes) of all annotated venom-related genes in the *P. flavoviridis* genome are located on microchromosomes (Fig. 3) [21,22]. This is the case for *C. tigris* as well, with all genes belonging to the major toxin family in the venom of this species (PLA_2_s) located on microchromosome 7 [33]. Phylogenetic analysis of the 3 most abundant and well-characterized toxin families in *C. viridis* venom (SVMPs, SVSPs, and PLA_2_s, all located on microchromosomes) revealed that each toxin gene family represents a distinct set of duplicated genes derived from a single ancestral homolog that produced a monophyletic cluster of venomous paraphyletic lineages [21]. Notably, microchromosomes have higher GC content and faster recombination rates than macrochromosomes [21], as is evident in the *C. viridis* genome [20]. Therefore, it appears that microchromosomes are generally enriched with venom genes, which together with their high recombination rate could explain the huge radiation and rapid evolution of venom-related genes [15].

Nonetheless, it should be noted that a substantial percentage of toxin-coding genes are found on macrochromosomes as well. This is evident in *N. naja*, where as many as 16 toxin gene families are located on macrochromosomes [32]. WGS of other venomous snake species will be essential to investigate how and to what extent chromosomal location of genes influences venom evolution. Interestingly, the chromosome structure of *C. viridis* is comparable to that of *N. naja*. In fact, chromosome 4 of *N. naja* shares syntenic regions with *C. viridis* chromosomes 3 and 5, and chromosomes 5 and 6 of *N. naja* are syntenic with chromosome 5 of *C. viridis* [27]. This might indicate the occurrence of fusion and fission events, respectively [27]. The *N. naja* genome has also been compared to that of *O. hannah* (another elapid, and thus more closely related to *N. naja* than *C. viridis*), where 139 venom gland toxin genes from the *N. naja* genome were cross-referenced with genes in the *O. hannah* genome to find orthologs [27]. The results showed that 96 of the *N. naja* genes had counterparts in the *O. hannah* genome, while 43 did not [27]. Although some of these 43 genes may be unique to *N. naja*, others may simply not have been annotated in the *O. hannah* genome, possibly owing to the high fragmentation of its assembly (which relied on short reads) [14].

In the future, widespread access to different types of sequencing platforms providing researchers with both short and long reads, complementary tools for genome analysis (Hi-C and CHiCAGO), and higher quality sequence data will likely enable researchers to study snake genomes in greater detail. In turn, this will help elucidate differences and similarities between snake genomes and allow for more fine-grained studies of the structural characteristics of snake venom genes.

### Molecular origin and regulation of snake venom genes

Snake venoms and their evolutionary origins have received substantial attention over the past decades, with >15,000 studies published on this topic [15]. Snake venoms have the dual functions of defense against predators and subduction of prey, with predation typically being the primary function [104]. This locks snakes and their prey in an evolutionary arms race, where the prey evolves biological strategies that make it resistant to toxins, and snakes are constantly pressured to optimize and adjust the composition of toxins in their venoms [104]. Indeed, dietary habits have often been indicated as a key driver of adaptive venom evolution in snakes, featuring among the main reasons behind inter- and intraspecific variation in venom composition [119].

Reports on trophic adaptations of snakes are plentiful. As an example, a study showing that venom variation in the Malayan pit viper (*Calloselasma rhodostoma*) throughout its range is significantly associated with the types of prey locally available [120]. This is also the case for the Mangrove catsnake (*Boiga dendrophila*), which was found to possess a 3FTx specific for birds and lizards (the bulk of this snake's diet) but scarcely effective on mammals [121]. However, recent research reported that venom composition in the Mojave rattlesnake (*Crotalus scutulatus*) was associated with environmental factors (e.g., temperature, seasonality) rather than with diet [122]. This suggests that a more complex scenario of factors could be affecting venom diversity than prey-related drivers alone, as confirmed by the dynamics behind venom variation in the Northern Pacific rattlesnake (*Crotalus oreganus*). In fact, the dichotomy in venom composition observed in this species is consistently influenced not only by coevolution with its prey but also by genetic distance and elevation-based habitat gradients, in a pattern described as “phenotype matching” of venom characteristics to multiple variables in the snake's native ecosystem [123,124]. The genetic basis underlying such complex adaptive processes could likely provide intriguing insight into the influence of natural selection and phylogenetic relatedness on the evolution of a highly dynamic trait such as snake venom. To this end, WGS of snakes will likely be key to conclusively determining the structural and evolutionary features of toxin genes and gene clusters. Analysing such patterns in a comparative framework would then enable researchers to identify similarities and differences in adaptive drivers of venom evolution at all levels of snake taxonomy and phylogeny.

In recent years, venom evolution has been further explored through genome studies on several species of venomous snakes [15,21,27,86]. One of these studies revealed that the venom gene repertoire of *D. acutus* has a very different composition from those of *O. hannah* and the non-venomous *A. carolinensis* (outgroup)*, B. constrictor*, and *P. bivittatus*. These differences are exemplified both by the absence of characteristic venom genes from the *D. acutus* genome relative to the *O. hannah* genome and by the increased gene copy number of other venom gene families, including SVMPs, CTLPs, and SVSPs (Table 1) [86]. Expression of most toxin-encoding genes shared by *D. acutus* and *O. hannah* (especially older genes derived from the last common ancestor of these species) is limited to venom glands or accessory glands [86]. Similarly, newer viper-specific toxin genes are expressed in the venom and accessory glands of *D. acutus*, while equally recent elapid-specific toxin genes are expressed in the venom and accessory glands of *O. hannah* [86]. Interestingly, genes closely related to the elapid-specific toxin genes expressed in the venom glands of *O. hannah* are expressed in the liver of *D. acutus*, and genes related to viper-specific toxin genes expressed in the venom glands of *D. acutus* are expressed in pooled organs from *O. hannah* [86].

These special expression patterns suggest that these venom genes may originate from metabolic proteins that have undergone subfunctionalization (i.e., paralogs retaining only part of the functional features of the original gene following duplication) or neofunctionalization, as well as that changes in tissue-specific expression have occurred [17, 86]. This is in accordance with previous protein-based findings [125,126]. Similarly, analysis of the *O. hannah* genome demonstrated that the regulatory components of the venomous secretion system may have evolved from the pancreas [18]. Several mechanisms likely contribute to the enhanced expression of toxin-coding genes in the venom gland. At the chromosome level, methylation and chromatin accessibility were recently shown to play a prominent role in gene regulation in *C. tigris*. In fact, methylation appears to be significantly more prevalent in non-toxin and unexpressed toxin genes compared to expressed toxin counterparts in the venom gland and pancreas of this species [33]. Furthermore, chromatin accessibility and methylation levels are positively related with the high expression of toxin genes compared to non-expressed counterparts and non-toxin genes in *C. tigris*, further supporting a joint role for these 2 factors in toxin gene expression [28]. Another important factor in regulation and expression of toxin genes is the gene regulatory network associated with them (recently termed “metavenom network”), which comprises ∼3,000 genes that do not code for toxins but actively influence their expression and postgenomic modifications (e.g., protein folding) in the venom gland as housekeeping genes [48]. Interestingly, this network presents highly conserved elements common to even distantly related lineages such as snakes and venomous mammals; on the other hand, snakes (specifically *P. flavoviridis* and *P. mucrosquamatus*) also displayed several unique regulatory genes that were likely co-opted together with neofunctionalized toxin genes absent in other lineages [48].

**Figure 4:**
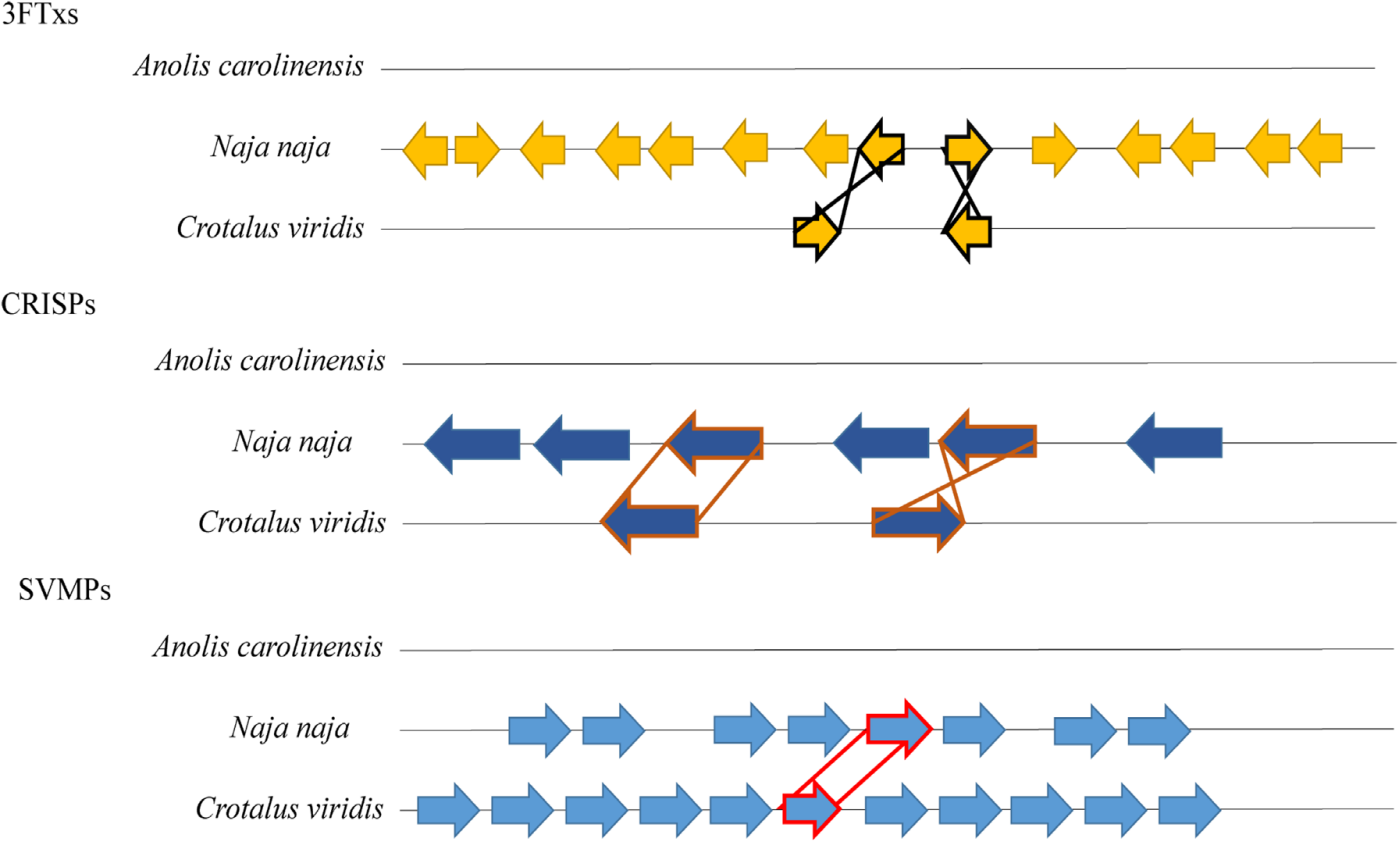
Syntenic comparison of toxin gene clusters. Comparison showing the 3FTx, CRISP, and SVMP genes in *N. naja* and *C. viridis* genomes. Orthologous gene pairs are indicated by the line linked across the corresponding genomic regions. Based on Fig. 4 and Extended Fig. 4 from [27].

Gene duplication is thought to be one of the main mechanisms behind venom diversification [127]. The current consensus is that 2 rounds of whole-genome duplication (2R-WGD) occurred during the evolution of vertebrates [15,128]. A study of the *P. flavoviridis* genome identified 18 families of venom-related genes, including both toxin and non-toxin gene copies. These include metalloproteinases (SVMPs), serine proteases (SVSPs), CTLPs, PLA_2_s, 3FTxs, aminopeptidases (APaseNs), CRISPs, vespryns/SPla and ryanodine receptor domain proteins (Vespryns), 5′-nucleotidases (5Nases), dipeptidyl peptidases (DDPases), hyaluronidases (Hyals), nerve growth factors or neurotrophins (NGF), vascular endothelial growth factors (VEGFs), LAAOs, phosphodiesterases (PDEs), phospholipases B (PLBs), bradykinin-potentiating peptides and C-type natriuretic peptides (BNPs), and glutaminyl peptide cyclotransferases (GPCases). [15]. The study suggested that 2R-WGD resulted in the creation of 4 paralogs from each of the 18 genes and that during the later evolution of venomous snakes, 1 of these 4 gene copies underwent neo- or subfunctionalization and evolved toxic properties, while the remaining 3 copies did not [15]. Both the toxin and non-toxin encoding genes subsequently underwent multiplication to different extents (Fig. 4) [15], as is demonstrated by the multiple gene duplication events detected in the SVMP, SVSP, CTLP, PLA_2_, 3FTx, and CRISP gene families in *P. flavoviridis* and *N. naja* [15,27]. However, this phenomenon was investigated to the greatest detail in rattlesnakes (*Crotalus* spp.), with comparative genomics between species revealing multiple duplication events in neurotoxic PLA_2_ genes as well as all SVMP classes. Chromosome mapping of the complete genomes of *C. viridis* and *C. tigris* provided further support for the occurrence of this phenomenon, highlighting similar duplication events for both gene families as well as SVSP genes (all of which are arranged in tandem-array single clusters) [18, 26].

Molecular phylogenetic analysis of *P. flavoviridis* shows that all toxin genes of a given gene family in this species are homologous to the same toxin gene families found in vipers and elapids, such as *P. mucrosquamatus* (brown-spotted pit viper) and *O. hannah* [15]. The notion that snake toxin genes massively expanded through gene duplication events and underwent sub- and/or neofunctionalization is also supported by other studies [18,27,108]. For example, the *N. naja* genome assembly contributes to our understanding of the origin of multiple unlinked venom gene clusters and provides new and conclusive evidence that each toxin family stems from a unique set of tandem duplicate genes [27].

While duplication either before or after gene recruitment to the venom gland is an established driving force of venom evolution in snakes, loss of genetic material has been no less pivotal in facilitating diversification of toxin families in certain venomous snake clades. For instance, the interplay between gene duplication and deletion (of entire genes as well as intragenic regions) is remarkable in rattlesnakes (*Crotalus* spp.). These pit vipers present signs of multiple independent losses of ancestral genes coding for SVMPs and neurotoxic PLA_2_s—both of which had previously experienced a rampant expansion via repeated duplication episodes—across their phylogenetic tree [39, 71]. Intriguingly, different genes underwent deletion among and even within species, such as observed in the western diamondback rattlesnake (*Crotalus atrox*), the Mojave rattlesnake (*C. scutulatus*), and the Southern Pacific rattlesnake (*Crotalus helleri*) [39,64,71]. This resulted in great haplotype disparity and differential expression of toxin-encoding genes not only between species but across conspecific individuals as well. WGS of *C. tigris* further corroborated this pattern, as this species is known for its remarkably simple venom composition largely based on neurotoxic PLA_2_ isoforms [129]. However, the *C. tigris* genome revealed a deletion of 3 PLA_2_ genes on microchromosome 7 and of 10 SVMP genes on microchromosome 1 compared to homologous regions in *C. viridis*, indicating that even such a simple venom phenotype is the result of extensive genomic modifications over evolutionary time [28]. This pattern is not limited to rattlesnakes. For instance, the *B. jararaca* genome also displays a great expansion of SVMP genes via duplication upon recruitment in the venom gland, followed by 2 deletions in the exon 14 region of PII-SVMP genes causing loss of the Cys-rich domain found in PIII-SVMPs [29]. This observation sheds further light on the genomic processes responsible for evolution and differentiation via domain loss in SVMPs, which has occurred in other viper lineages as well [46].

### Adaptive and neutral evolution in snake venoms

Determining and unraveling the driving factors behind the dynamic evolutionary processes in snake venom gene families has garnered the interest of scientists for decades—a quest that could only benefit from increasing efforts in WGS of venomous snakes. Positive selection appears to be the force behind the evolution of genes involved in predator-prey arms races [130], and it seems to be pervasive across most toxin-related gene families in snakes. Positive selection leaves a well-defined pattern in the genome, with the accumulation of non-synonymous, amino acid–replacing nucleotide substitutions (denoted by K_a_), over synonymous substitutions (K_s_) in the gene [131]. In *P. flavoviridis*, the K_a_/K_s_ ratios of the 4 main toxin gene families were consistently > 1 and/or higher than those reported for non-venom genes (SVMPs: 1.047 ± 0.438, SVSPs: 1.253 ± 0.090, CTLPs: 0.871 ± 0.071, PLA_2_s: 1.093 ± 0.062) [15], suggesting positive selection behind the accelerated evolution of the major toxin gene families in this species. Interestingly, *P. flavoviridis* also exhibited K_a_/K_s_ > 1, in the 3FTx and CRISP gene families, which therefore also displayed a tendency towards accelerated evolution despite being present in far fewer copies [15]. Similarly, a high K_a_/K_s_ ratio (2.034 ± 0.818) was observed for the 3FTx gene family in the *N. naja* genome, again pointing towards rapid differentiation and functional diversification for these genes [27]. Conversely, when K_a_/K_s_ < 1 it is indicative of either neutral selection (random substitutions that confer neither evolutionary advantages nor disadvantages) or purifying selection (i.e., removal of mutations that usually tend to be deleterious as they appear in conserved areas of the gene). In the *P. flavoviridis* genome study, all non-dominant toxin gene families had a K_a_/K_s_ < 1 (mean = 0.512 [SD 0.018]), indicating a more neutral nucleotide substitution and the maintenance of similarity between gene copies [15]. On the other hand, when examining sequence divergence using venom gland transcriptomes in sidewinder rattlesnakes (*Crotalus cerastes*), data showed evidence of selection being stabilized, which supports that the maintainance of a generalist phenotype is favoured [132]. It must, however, be noted that despite various methods available for studying selection (see [133]), relatively few have been applied for the investigation of selection in venom and only in a small number of species [15,27, 132, 134]. Therefore, additional studies are required before general conclusions can be drawn.

New -omics tools and methods are rapidly advancing our knowledge of the mechanisms behind venom evolution [135]. In particular, WGS has introduced advantages to snake venom research, as WGS data can be used to identify structural variants, including inversions (Fig. 3A-B), insertions (Fig. 3C), deletions, tandem repeats (Fig. 3A-C), TEs, and other repeat content [21, 136]. An increasing number of studies report venom variation at different levels, such as ontogenetic, within-species, and between-species [46,131,137–140]. Once the reference genome of a species is available, population genomics can contribute to the identification of such intra- or interspecific variation. This further enhances the study of venom regulation, helping us to understand the evolution of complex regulatory networks [28]. Although it is generally acknowledged that positive selection seems to be the main driver behind venom evolution, genomic tools allow zooming in on specific venom-related genes to infer the role of neutral evolutionary processes, i.e., genetic drift or random changes in allele frequencies [141]. Genetic drift contributes to the accumulation of random neutral variation, which serves as the basis for natural selection to act upon in response to new evolutionary pressures [142]. Although most research to date has focused on the adaptive processes explaining venom evolution, recent studies have started assessing the role of such neutral forces in shaping venom characteristics. For example, genetic drift was identified as a prominent factor behind sequence divergence in venom genes in *P. mucrosquamatus*, where dominant toxin-encoding genes displayed relaxed selective constraints for deleterious mutations despite statistically significant rates of positive selection [24]. Furthermore, it has been shown that variation in expression of the myotoxin, crotamine, in the eastern diamondback rattlesnake (*Crotalus adamanteus*) and the South American rattlesnake (*Crotalus durissus*) is significantly more correlated with differences in number of duplication-derived gene copies between populations than with adaptive divergence in the sequences themselves [134,143].

The strength at which genetic drift acts on the genome is inversely proportional to effective population size (*N_e_*, namely, the number of reproductive individuals that actually produce offspring) [142]. *N_e_* greatly contributes to sequence variation, as the fate of a favourable mutation spreading is controlled by *N_e_* and the strength of selection [144,145]. A prime example of this pattern in snake venom evolution is presented by the eastern massasauga rattlesnake (*Sistrurus catenatus*), a threatened species whose range consists of several scattered populations largely isolated from each other. Although the influence of genetic drift on venom evolution in this species is currently weak, it is likely to increase dramatically over time once the impact of drift is augmented due to the low *N_e_* found in most populations [146]. Thus, complete genomes obtained through WGS together with complementary DNA libraries can expand our knowledge of the effects of selection on venom genes, with great potential to either corroborate or challenge the current positive selection–centered view of snake venom evolution.

## Conclusions and Perspectives

WGS is a revolutionary advance in genetic research that has only recently been applied to the fields of herpetology and toxinology. Nonetheless, sequencing of complete snake genomes has already shed light on the evolutionary history of toxin-encoding genes as well as their expression patterns in the venom gland. In the future, WGS may be harnessed to obtain a better understanding of the molecular mechanisms involved in snake evolution [6,103], find new bioactive molecules with potential clinical applications, and provide valuable information for antivenom development [35]. Because only 21 complete snake genomes are currently available, there is ample opportunity for genomic research on the remaining thousands of snake species, including medically relevant venomous representatives. With the increasing power of sequencing technologies, the field of snake genomics is indeed likely to expand significantly in the years to come, with multiple complete genomes already in the process of being sequenced or published. However, this will not come without challenges because the interplay of dietary and environmental factors that has fueled venom diversification via gene duplication, recruitment, and neofunctionalization events makes it difficult to assemble whole venomous snake genomes. Another factor adding to the complexity of *de novo* genome assembly is the high content of repeat sequences in snake genomes. Some of these challenges might be adequately addressed by using third-generation sequencing technology. As the costs and error rates of this and other approaches decrease, they are certain to be used more widely in snake genome research. In turn, the assembly of more venomous snake genomes will allow us to explore adaptation and venom evolution at all phylogenetic levels, bringing a new perspective to the study of snake genomes and venoms.

## Data Availability

Not applicable.

## Abbreviations

3FTx: 3-finger toxin; bp: base pairs; BUSCO: Benchmarking Universal Single-Copy Orthologues; CRISP: cysteine-rich secretory protein; CTLP: C-type lectin-like protein; DoC: depth of coverage; Gb: gigabase pairs; kb: kilobase pairs; LAAO: L-amino acid oxidase; LINE: long interspersed element; NCBI: National Center for Biotechnology Information; NGS: next-generation sequencing; PLA_2_: phospholipase A_2_; SSR: simple sequence repeat; SVMP: snake venom metalloproteinase; SVSP: snake venom serine proteinase; TE: transposable element; WGS: whole-genome sequencing.

## Competing Interests

W.R., W.Z., and S.L. are employees at the BGI. The authors declare that they have no other competing interests.

## Funding

This research was supported by the Beijing Genomics Institute and the Technical University of Denmark. M.E.A. is funded by the Independent Research Fund Denmark (7027–00147B). C.K. is funded by Innovation Fund Denmark (9065–00007B). T.P.J. is funded under Marie Sklodowska-Curie grant agreement No. 713683 (COFUNDfellowsDTU).

## Authors’ Contributions

A.H.L. and S.L. conceived the project. A.H.L., W.R., K.K., T.P.J., C.K., C.T.W., W.Z., S.G., L.S., M.M.D., B.J.M., E.R.T., and M.E.A. structured the draft and provided final editing. A.H.L., K.K., T.P.J., and W.R. coordinated and drafted the manuscript and implemented comments provided by all authors. All authors contributed critically to the scientific content. All authors read and approved the final manuscript.

## Supplementary Material

giac024_GIGA-D-21-00410_Original_Submission

giac024_GIGA-D-21-00410_Revision_1

giac024_Response_to_Reviewer_Comments_Revision_1

giac024_Reviewer_1_Report_Original_Submission Blair Perry -- 1/18/2022 Reviewed

giac024_Reviewer_2_Report_Original_Submission Matthew Holding -- 1/18/2022 Reviewed
