# Supplementary material for: The rise of genomics in snake venom research: recent advances and future perspectives
Source: Gigascience. 2022 Apr 1;11:giac024. doi: 10.1093/gigascience/giac024 (PMC8975721; doi:10.1093/gigascience/giac024)

GigaScience
The rise of genomics in snake venom research: recent advances and future perspectives
--Manuscript Draft--

**Manuscript Number:**	GIGA-D-21-00410
**Full Title:**	The rise of genomics in snake venom research: recent advances and future perspectives
**Article Type:**	Review
**Funding Information:**	Danmarks Frie Forskningsfond (7027-00147B) — Not applicable<br>Innovationsfonden (9065-00007B) — Not applicable<br>COFUNDfellowsDTU (713683) — Not applicable
**Abstract:**	Snake venoms represent a danger to human health, but also a goldmine of bioactive proteins that can be harnessed for drug discovery purposes. The evolution of snakes and their venom has been studied for decades, particularly via traditional morphological and basic genetic methods alongside venom proteomics. However, while the field of genomics has matured rapidly over the past two decades due to the development of Next Generation Sequencing (NGS) technologies, snake genomics remains in its infancy. Here, we provide an overview of the state-of-the-art in snake genomics and discuss its potential implications for studying venom evolution and toxinology. Based on current knowledge, gene duplication and positive selection are key mechanisms in the neofunctionalization of snake venom proteins. This makes snake venoms important evolutionary drivers that explain the remarkable venom diversification and adaptative variation observed in these reptiles. Gene duplication and neofunctionalization have also generated a large number of repeat sequences in snake genomes that pose a significant challenge to DNA sequencing, resulting in the need for substantial computational resources and longer sequencing read length for high quality genome assembly. Fortunately, owing to constantly improving sequencing technologies and computational tools, we are now able to explore the molecular mechanisms of snake venom evolution in unprecedented detail. Such novel insights have the potential to impact the design and development of antivenoms and possibly other drugs, as well as provide new fundamental knowledge on snake biology and evolution.
**Corresponding Author:**	Andreas Hougaard Laustsen<br>Technical University of Denmark<br>Kongens Lyngby, DENMARK
**Corresponding Author Secondary Information:**	
**Corresponding Author's Institution:**	Technical University of Denmark
**Corresponding Author's Secondary Institution:**	
**First Author:**	Wei-qiao Rao
**First Author Secondary Information:**	
**Order of Authors:**	Wei-qiao Rao<br>Konstantinos Kalogeropoulos<br>Morten E. Allentoft<br>Shyam Gopalakrishnan<br>Wei-ning Zhao<br>Christopher T. Workman

Powered by Editorial Manager® and ProduXion Manager® from Aries Systems Corporation

	Cecilie Knudsen
	Belén Jiménez-Mena
	Lorenzo Seneci
	Mahsa Mousavi-Derazmahalleh
	Timothy P. Jenkins
	Si-qi Liu
	Andreas Hougaard Laustsen
	Esperanza Rivera-de-Torre
**Order of Authors Secondary Information:**	
**Additional Information:**	
**Question**	**Response**
Are you submitting this manuscript to a special series or article collection?	No
**Experimental design and statistics**<br><br>Full details of the experimental design and statistical methods used should be given in the Methods section, as detailed in our Minimum Standards Reporting Checklist. Information essential to interpreting the data presented should be made available in the figure legends.<br><br>Have you included all the information requested in your manuscript?	Yes
**Resources**<br><br>A description of all resources used, including antibodies, cell lines, animals and software tools, with enough information to allow them to be uniquely identified, should be included in the Methods section. Authors are strongly encouraged to cite Research Resource Identifiers (RRIDs) for antibodies, model organisms and tools, where possible.<br><br>Have you included the information requested as detailed in our Minimum Standards Reporting Checklist?	Yes
**Availability of data and materials**	Yes

All datasets and code on which the conclusions of the paper rely must be either included in your submission or deposited in publicly available repositories (where available and ethically appropriate), referencing such data using a unique identifier in the references and in the "Availability of Data and Materials" section of your manuscript.<br><br>Have you have met the above requirement as detailed in our Minimum Standards Reporting Checklist?	

Manuscript

Click here to access/download;Manuscript;2021-12-13 Rao et al. Snake genomics v16AHL.docx

The rise of genomics in snake venom research: recent advances and future perspectives

Wei-qiao Rao[1,2], Konstantinos Kalogeropoulos[1], Morten E. Allentoft[3,4], Shyam Gopalakrishnan[4], Wei-ning Zhao[2], Christopher T. Workman[1], Cecilie Knudsen[1], Belén Jiménez-Mena[5], Lorenzo Seneci[1], Mahsa Mousavi-Derazmahalleh[3], Timothy P. Jenkins[1], Si-qi Liu[2*], Andreas H. Laustsen[1*]

[1]Department of Biotechnology and Biomedicine, Technical University of Denmark, Kongens Lyngby, Denmark
[2]Department of Mass Spectrometry, Beijing Genomics Institute-Research, Shenzhen, China
[3]Trace and Environmental DNA (TrEnD) Laboratory, School of Molecular and Life Sciences, Curtin University, Perth, Australia
[4]Globe Institute, University of Copenhagen, Copenhagen, Denmark
[5]DTU Aqua, Technical University of Denmark, Silkeborg, Denmark

Address for correspondence:

Dr. Siqi Liu
Professor
Department of Mass Spectrometry,
Beijing Genomics Institute-Research
Shenzhen, China
*siqiliu@genomics.cn

Dr. Andreas H. Laustsen
Professor
Department of Biotechnology and Biomedicine
Technical University of Denmark
DK-2800 Kongens Lyngby, Denmark
*ahola@bio.dtu.dk

Abstract

Snake venoms represent a danger to human health, but also a goldmine of bioactive proteins that can be harnessed for drug discovery purposes. The evolution of snakes and their venom has been studied for decades, particularly via traditional morphological and basic genetic methods alongside venom proteomics. However, while the field of genomics has matured rapidly over the past two decades due to the development of Next Generation Sequencing (NGS) technologies, snake genomics remains in its infancy. Here, we provide an overview of the state-of-the-art in snake genomics and discuss its potential implications for studying venom evolution and toxinology. Based on current knowledge, gene duplication and positive selection are key mechanisms in the neofunctionalization of snake venom proteins. This makes snake venoms important evolutionary drivers that explain the remarkable venom diversification and adaptative variation observed in these reptiles. Gene duplication and neofunctionalization have also generated a large number of repeat sequences in snake genomes that pose a significant challenge to DNA sequencing, resulting in the need for substantial computational resources and longer sequencing read length for high quality genome assembly. Fortunately, owing to constantly improving sequencing technologies and computational tools, we are now able to explore the molecular mechanisms of snake venom evolution in unprecedented detail. Such novel insights have the potential to impact the design and development of antivenoms and possibly other drugs, as well as provide new fundamental knowledge on snake biology and evolution.

Keywords: Snake genomics; DNA sequencing; venom; venom evolution; snakes; snake toxins

1. Background

Snakes (Squamata: Serpentes) represent a monophyletic lineage, comprising approximately 3,600 extant species found in all continents, except Antarctica [1,2]. From an evolutionary perspective, these reptiles stand out for their characteristic lack of limbs, elongated body shape, and exclusively carnivorous diet. Even before the advent of genetic approaches, conventional anatomical and morphology-based phylogenetic evidence unambiguously suggested that snakes evolved from lizards [3]. Together with amphisbaenians, these two groups form the largest branch of terrestrial vertebrates, the squamate reptiles [3]. Snakes have many specialized adaptations compared to other reptile lineages. For example, the evolution of infrared sensing pits in pitvipers (Viperidae: Crotalinae), boas (Boidae), and pythons (Pythonidae), and of a venom apparatus in several snake families (Fig. 1), provide these animals with exceptional predatory capabilities despite the loss of limbs and the degradation of visual and auditory perception in many (but not all) species [4–6]. Moreover, severe jaw modifications and low metabolic rates enable snakes to swallow and digest large prey whole, further consolidating their position as formidable predators [7,8]. Thus, snakes are important model organisms for evolutionary studies, and have yielded insights into limb development [9–11], sex chromosome evolution [12], and venom evolution [13].

Fig. 1. *Schematic diagram of snake evolution based on data from Snakedatabase.org* [14]. *Snakes (Serpentes) are divided into the three main infraorders: Scolecophidia, Henophidia, and Alethinophidia, which combined encompass approximately 24 families (7 shown here). Families comprising venomous species have been marked with a skull and crossbones symbol. Colubridae constitutes the largest family of snakes, encompassing 52% of the approximately 3,566 snake species currently described. The total number of currently described venomous snake species (2,901), which predominantly fall within the families Homalopsidae, Lamprophiidae, Colubridae, Elapidae, and Viperidae. Only snake species that have undergone whole-genome sequencing and assembly are listed in this figure.*

The development of next-generation sequencing (NGS) technologies in recent decades has allowed researchers to generate large genomic datasets and rendered the assembly and characterization of complete genomes a routine task. Despite the availability of NGS since the early 2000s, the utilization of these technologies to assemble and study complete snake genomes has been very limited, especially when compared to the amount of research that has been conducted in the fields of snake venom proteomics and transcriptomics [15]. It was not until 2013 that the first snake genomes based on high-throughput sequencing data were published for the Burmese python (*Python bivittatus*), the Red-tailed boa (*Boa constrictor constrictor*), and the King cobra (*Ophiophagus hannah*) [7,16,17]. Fortunately, snake genome research has eventually gained more attention, with 18 new genomes being released since 2013 and several more currently in progress [13,18–29]. These increased sequencing efforts have already revealed intriguing insights into the regulation and expression of venom-related genes. As an example, a large number of dormant toxin-encoding genes with unknown bioactivity were identified in the Okinawan habu (*Protobothrops flavoviridis*) [13]. Such discoveries could be of high scientific value and may improve our basic understanding of the interplay between protein function and evolution. Furthermore, as toxins from several animal lineages are known to possess different types of bioactivity, some of them could find utility in a variety of applications, from the development of novel therapeutics [30] to biopesticides [31] and molecular research tools [32]. With only 21 snake genomes publicly available to date, there is great unexplored scientific potential in sequencing and analyzing more snake genomes [15,33].

From a broader perspective, having access to a complete or nearly complete assembled genome provides an excellent basis for addressing a wide range of biological research questions. For example, genomic data can be used to predict protein-coding exons [34] (including exons in genes that recently underwent pseudogenization), non-expressed genes, translated proteins, and microRNA (miRNA) genes [35]. Genomic data may also allow for the identification of toxin orthologs using

comparative studies and homology searches [36]. Knowledge of homology is crucial for the reliability of functional annotation of genomes and can provide fundamental information on evolution and speciation processes [37,38]. Therefore, complete genomes are crucial to the field of proteomics as well, as the absence of reliable genome-derived protein libraries forces researchers to rely on homologous proteins from other organisms as a benchmark to compare newly characterized protein sequences against. This results in severely limited accuracy in identifying potentially homologous proteins, which consequently leads to overlooking and/or misrepresenting evolutionary patterns. This is especially relevant considering the likely widespread occurrence of alternative splicing in snake genomes, which gives rise to multiple mRNA products that in turn result in various isoform of a particular toxin [39–41]. Extensive post-genomic and post-translational modifications are also at play, leading to often remarkable discrepancies between genome, transcriptome, and proteome in terms of expression and sequence identity [42–44]. Along this line, comparative analysis of whole snake genomes could likely provide invaluable insight on the evolution and structure of the gene regulatory network responsible for the expression of venom genes in these animals (and arguably venomous amniotes in general) [45].

Several approaches are available to obtain reliable genomic data. Among them is reduced-representation sequencing (RRS), in which only a part of the genome is sequenced [46]. Conversely, Restriction-Site Associated DNA sequencing (RAD-seq) uses restriction enzymes to obtain genome-wide sequencing data, or capture sequencing, which allows specific areas of interest (e.g., the exon part of the genome) to be targeted and sequenced [47]. Nevertheless, reliable detection of homologous genes across species and/or lineages can be hindered by the acquisition, loss, or pseudogenization of genes [38]. One way to overcome this challenge is to use whole-genome sequencing (WGS), which represents a more comprehensive resource for the detection of homologous genes as it provides the entire genotype of the target organism(s) [38]. WGS can also provide

information on genomic variability of a species, and potentially discover and quantify the extent of selective (e.g., positive/purifying selection and hitchhiking effects) and neutral forces (e.g., genetic drift) driving venom evolution [48].

This review aims to provide a comprehensive summary of the current knowledge on snake genomics, with a particular focus on the current use and future potential of high-throughput DNA sequencing technologies in the field of snake toxinology. Moreover, we discuss how these technologies can be used to expand our current knowledge on snake venom evolution and toxin diversification.

2. Current status of snake venom research

2.1 Overview of snake toxin families

Studies have estimated that between 19,000 and 25,000 toxins are found in venoms from the Elapidae and Viperidae snake families, but only a few thousands have been characterized [49]. Nonetheless, this body of knowledge has proven sufficient for the systematic classification of snake venom toxins into 63 families, most of which are, however, only found in a small percentage of snake species and/or in negligible amounts within venom mixtures. [50]. The four families generally considered to be of highest relevance both from a clinical (human envenoming cases) and an ecological perspective (e.g. prey incapacitation) are the three-finger toxins (3FTxs), phospholipases A_2 (PLA_2s), snake venom metalloproteinases ($SVMP_S$), and snake venom serine proteinases ($SVSP_S$). Other widespread snake venom protein families include Cysteine-rich secretory proteins (CRISPs), L-amino acid oxidases (LAAOs), and C-type lectin-like proteins (CTLPs) [50]. An overview of the main snake venom toxin families is provided in Table 1.

Table 1. Number of toxin-encoding genes for 22 toxin families in selected snakes.

Venom protein family	Venom family abbreviation	*Anolis carolinensis**	*Boa constrictor*	*Python bivittatus*	*Deinagkistrodon acutus*	*Ophiophagus hannah*
5’-nucleotidases	5Nase	1	1	1	1	1
Acetylcholinesterase	ACeH	22	11	12	14	16
Bovine pancreatic trypsin inhibitors	BPTI	86	39	49	70	53
Bradykinin-potentiating peptides and C-type natriuretic peptides	BNP	1	3	1	2	6
Cysteine-rich secretory proteins	CRISPs	2	1	1	2	3
C-type lectin-like proteins	CTLPs	5	7	6	22	13
Disintegrins	Dis	-	-	-	3	-
Factor V	-	5	5	6	5	5
Factor X	-	9	11	11	11	11
Hyaluronidases	HYAL	5	6	6	6	6
L-amino acid oxidases	LAAO	4	5	6	4	3
Nerve growth factors or neurotrophins	NGF	5	5	5	4	5
Ohanin-like proteins	-	24	12	9	14	9
Phosphodiesterases	PDE	6	6	5	5	5
Phospholipases A_2	PLA_2	1	1	1	1	4
Phospholipases B	PLB	1	1	1	1	4
Snake venom metalloproteinases	SVMP (PI)	-	2	-	1	-
	SVMP (PII)	-	1	-	4	-
	SVMP (PIII)	1	1	2	5	4
Snake venom serine proteinases	SVSP	4	6	7	22	8
Three-finger toxins	3FTx	-	-	-	-	5
Vascular endothelial growth factors	VEGF	4	7	7	6	5
Venom ficolins	Veficolins	11	9	9	10	11
Vespryns	-	66	28	43	28	30
Waprin	-	5	3	3	3	4

*The green anole (*Anolis carolinensis*) was selected as outgroup taxon as it is a non-venomous, non-snake squamate with a complete genome sequence available.

3FTxs belong to a superfamily of non-enzymatic proteins and are a major component in the venoms of most elapids, while they generally feature less prominently in viperid and colubrid venoms. These toxins have three β-stranded loops extending from a central core, contain four or five conserved disulfide bonds, and cause a wide range of pharmacological effects [51–53]. A prominent group of 3FTxs, α-neurotoxins, interfere with neuromuscular signal transmission of cholinergic

neurons by binding to nicotinic acetylcholine receptors, causing flaccid paralysis [53, 55]. Other 3FTxs are toxic to cardiomyocytes and can lead to increased heart rate and ultimately cardiac arrest, while yet others function as calcium channel blockers or platelet aggregation inhibitors [51].

PLA_2s are found in the venoms of vipers, elapids, and certain rear-fanged species [54–57] and exert a wide variety of cytotoxic, myotoxic, cardiotoxic, and neurotoxic effects [54,55,57]. Of particular interest is a catalytically inactive, myotoxic category of PLA_2s stemming from a single substitution of a highly conserved amino acid residue (Asp49 to Lys49/Asn49) [54]. Both non-catalytic and enzymatic PLA_2s are able to form heterodimeric complexes with other PLA_2s or other toxins in certain venoms, whereby their toxicity is greatly potentiated [55]. Most snake genomes contain multiple PLA_2 genes, which likely originated from repeated gene duplication events [57,58]. These paralogs have diverse pharmacological activities, which were likely acquired through neofunctionalization (i.e. recruitment of a paralog to the venom gland following gene duplication and its subsequent evolution into a toxin-coding gene) [59,60]. Pseudogenization and deletion of PLA_2 genes are also frequent in snakes, making this toxin family one of the most dynamic in terms of evolutionary history [26,37,61]. The annotation of more snake genomes, and the likely consequent discovery of more PLA_2 genes, might provide an improved understanding of the evolution and the mechanisms of action of these proteins (including how the phenomenon of toxin synergism has evolved), and potentially assist in the characterization of similar evolutionary processes for other enzymes.

Another major category of enzymes found in snake venoms are SVMPs [62,63]. These proteinases are enzymes that cleave peptide bonds in other proteins, which may result in the degradation or activation of the target [63]. Zinc-dependent SVMPs are often the major venom component in vipers [64], and these toxins hydrolyze extracellular matrix components, leading to

rupture of capillaries and local and systemic bleeding [56]. Other clinical manifestation induced by SVMPs include edema, inflammation, myonecrosis, and reduced muscle regeneration [64]. Additionally, these enzymatic toxins can have anticoagulant, clotting factor-activating, or platelet-aggregating effects [65,66]. SVMPs are divided into three distinct classes depending on the domains present in the mature enzymes: P-I (metalloproteinase (M) domain only), P-II (M domain and disintegrin-like domain), and P-III (M domain, disintegrin-like domain, and cysteine-rich domain) [62]. Elucidation of snake genomes could help shed light on how these enzymes evolved from the ancestral P-III type via loss of domains [67–69] and postgenomic modifications, acquiring different functions and specificities in the process [62]. A better understanding of SVMP evolution via snake genomics could also provide insight into the evolutionary process that led to the diversification of SVMPs as a whole from the ancestral A Disintegrin and Metalloprotease (ADAM) family of metalloproteinases, which play significant roles in all stages of development and survival of higher-order organisms [70].

Finally, SVSPs are typically present in the venoms of vipers [71], but can also be found in elapid venoms [72]. SVSPs contain two six-stranded β-barrels and consist of approximately 245 amino acid residues. SVSPs also have a unique extended C-terminus that forms a disulfide bridge, which contributes to structural stability [73]. These toxins can induce blood coagulation through fibrin formation, Factor V activation, prothrombin activation, actin dissolvement, or platelet aggregation; conversely, they can also act as anticoagulants via fibrinolysis, fibrinolytic enzyme activation, or protein C activation [56,74–76]. This toxin family has received increased attention with recent genome studies on *P. flavoviridis* and *B. jararaca,* where the evolutionary pathway as well as the molecular regulation of SVSP expression was systematically investigated [13,27].

In summary, snake toxin families are numerous and their pharmacological actions are complex [77]. Knowledge on the toxicity and structure of different snake toxin families is essential to

further our understanding of snake venom evolution, as well as to understand venoms as drug targets for antivenom development. Much knowledge has been gained from venom proteomics and transcriptomics, and new genomics technologies now allow for the investigation of the evolutionary relationships between toxins in different families in unprecedented detail.

2.2 State-of-the art in snake genomics

With the rapid development of high-throughput sequencing technology, large-scale genomic projects have generated rich sequence information data of billions of base pairs and have paved the way for a new era in the field of phylogenetics, whereby the evolutionary history of organisms can be reconstructed from genomic data. The supermatrix method is the most well-known approach for analyzing concatenation of multiple gene sequences, and using genomic data sets with improved resolution can potentially mitigate phylogenetic problems previously caused by sampling errors [78]. However, since only 21 (approximately 0.6%) out of the ca. 3,600 existing snake species have undergone WGS so far [7,13,15,16,18–26,28,79–82], snake genomics will likely develop significantly in the coming years. A complete list of currently available snake genomes is provided below in Table 2.

INSERT TABLE 2 HERE

Available snake genomes differ notably in their assembly and annotation qualities, which makes evaluation of genome quality an important factor in determining the suitability of a genome for addressing a given set of questions. For instance, while estimation of nucleotide composition and genomic repeat content can be achieved from a relatively fragmented genome assembly, high-quality genome assemblies are required for analyses of multi-gene families and regulatory elements [83]. Genome assembly quality is assessed using statistics that measure fragmentation of the genome

assembly, such as total assembly length, total contig number, contig N50, and scaffold N50. The total length of the assembly represents the total length of all the contigs that are part of the *de novo* assembled genome. A high total assembly length usually indicates a high-quality genome assembly. The contig N50 expresses the contiguity of the assembled genome. For instance, a contig N50 of 10 kilo bases (kb) implies that 50% of the entire genome assembly is contained in contigs that are longer than 10kb. Thus, a high contig N50 value represents a high-quality assembly without too many gaps. Currently, the contig N50 values of most published snake genomes are <25 kb; exceptions include seven species with better assembly quality, namely *Thamnophis elegans* (Western terrestrial garter snake; 4,620 kb), *Crotalus tigris* (Tiger rattlesnake; 2,110 kb) [32], *Naja naja* (Indian cobra; 304 kb) [31], *Hydrophis curtus* (Shaw's sea snake; 183 kb) [28], *Bothrops jararaca* (Brazilian lancehead; 163.5 kb) [27], *Pseudonaja textilis* (Eastern brown snake; 51 kb) [81], and *Notechis scutatus* (Tiger snake; 32 kb) [80].

Another important parameter is the contig L50, which represents the minimum number of contigs required to cover 50% of the total assembly length. N50 and L50 values can be computed both at the contig and scaffold level. The most complete published snake genomes to date are those of *N. naja* and *C. tigris*, which were assembled by combining data obtained from long-read platforms (PacBio and Nanopore) and short-read platforms (Illumina), as well as Chicago, Hi-C, and optical mapping in the case of *N. naja* [31, Fig. 2]. The resulting assemblies have a scaffold N50 reaching a staggering 207.72 Mb (*C. tigris*) and 223.35 Mb (*N. naja*) in length , which is roughly 2.5 times greater than the previously assembled human reference genome (87 Mb) [31, 32, 88].

Fig. 2 *Schematic representation of the next-generation sequencing (NGS) pipeline for genomic assembly.* ***(1)*** *multiple companies have marketed sequencing platforms for genomic and transcriptomic studies, the most commonly used being Illumina (left), PacBio (middle), and Nanopore*

(right). ***(2)*** *The three platforms differ in read length and accuracy of the their generated sequences. Whilst Illumina sequencing generally yields short reads with low error rates, Nanopore sequences are substantially longer (up to 2 Mb), yet subject to frequent sequencing errors. Lastly, PacBio generates sequences with lengths and error rates in between the two other platforms.* ***(3)*** *After sequencing, reads are computationally processed and assembled into contigs, which in turn* ***(4)*** *serve as the building blocks for scaffolds.* ***(5)*** *The scaffolds are then aligned and annotated to produce the complete target genome.*

In addition to measures of genome contiguity – such as N50 scores – evaluating the representation of genes in a genome assembly via tools such as Benchmarking Universal Single-Copy Orthologues (BUSCO) provides great insight into genome assembly and annotation completeness [85]. A recent study using 611 published eukaryotic genomes showed that assemblies with high contig and scaffold N50 scores were shown to have high BUSCO values as well. However, the study revealed that assemblies with poor N50 scores may also (albeit rarely) show high BUSCO scores [86]. One example of this scenario in snakes is the case of the *P. flavoviridis* genome assembly where contig N50 was 3.8 kb, but percentages of complete and partial coverages for a set of 233 core vertebrate genes were 92.7% and 97.0%, respectively [18].

Furthermore, much can be learned about the quality of a genome from its reported depth of coverage (DoC). A DoC of 10X implies that each position in the genome has been read on average 10 times from independent sequencing reads. High DoC values imply that each position (i.e. each nucleotide) can be determined with greater confidence. Consequently, the 21 snake genomes published to date or in progress can be categorized into two groups: (1) a high DoC group (>50X) comprising *B. jararaca* (Brazilian lancehead) [27], *Crotalus viridis* (Prairie rattlesnake) [18,19], *C. horridus* (Timber 0rattlesnake) [79], *P. flavoviridis* [13], *P. mucrosquamatus* (Brown-spotted pitviper) [22], *Vipera berus*

(European adder) [23], *Thermophis baileyi* (Tibetan hot-spring snake) [21], *Thamnophis sirtalis* (Common garter snake) [82], *T. elegans* (Western terrestrial garter snake), *P. textilis* [81], *C. tigris* (Tiger rattlesnake) [26], *N. scutatus* (Tiger snake) [87], *D. acutus* (ive-pacer viper) [88], *B. constrictor* (Red-tailed boa) [17], *H. curtus* (Shaw's sea snake) [28], and *N. naja* [25]; and (2) a low DoC group (13-40X), which includes *Sistrurus catenatus* (Eastern massasauga rattlesnake), *C. pyrrhus* (Southwestern speckled rattlesnake) [24], *Pantherophis guttatus* (corn snake) [20], *O. hannah* [16], and *Python bivittatus* [7]. Unsurprisingly, the earliest published snake genomes are characterized by lower DoCs, whereas the more recently sequenced genomes benefitted from technological advancement and thus generally obtained better coverages. The best example of this is the *N. naja* genome, which reached a DoC of 250X [25] – by far the highest DoC reported for a snake genome to date (Table 2). This high DoC enabled the discovery of 43 new toxin-encoding genes, some of which are likely to be unique to *N. naja* [25].

Genome size (the total amount of DNA contained within one copy of a single complete genome [89]), number of genes, and guanine-cytosine content (GC-content, the percentage of the two nitrogenous bases in DNA [90]) vary from species to species and therefore may help elucidate phylogenetic relationships and molecular events (e.g., gene/genome duplication, pseudogenization, gene loss) in the evolution of species. Genome size can vary greatly and is typically correlated with organism size and complexity as well as with genome repeat content [89]. The reported genome sizes of snakes range from 1.3 Gb to 1.8 Gb, except for *C. pyrrhus* (1.1 Gb) and *B. jararaca* (2.1 Gb) (Table 2). This is consistent with previous findings that squamate reptiles and birds generally have smaller genomes than mammals (1-3 Gb for squamates vs 1-2 Gb for birds vs 2-6 Gb for mammals) (Table 3) [22]. Somewhat counterintuitively, genome size is not necessarily correlated with the number of genes in the genome. For example, although the *H. sapiens* genome (2.90 Gb) is roughly two times larger than the *T. sirtalis* genome (1.42 Gb), the number of genes is similar between the two (20,186 genes

for *T. sirtalis* compared to 21,407 genes for *H. sapiens*) [91]. This is because even though the average gene length of *T. sirtalis* (13,384 bp) is significantly smaller than that of *H. sapiens* (23,247 bp), the lengths of the exons are comparable [91].

Unlike their genome sizes and gene lengths, the genomic GC-contents for mammals, birds, and squamates are similar (~40%) (Table 3), and the GC-contents of reported snake genomes range from 34.3% to 43.6% (Table 3). Interspecies variation in GC-content is thought to be caused by selective variation, mutation bias, and biased DNA repair-related recombination [90]. High GC-content might also be an indication of biased sequencing results [92]. It is advisable to obtain information regarding both genome size and GC-content prior to *de novo* assembly of a genome, as these key genomic features can guide the choice of the most appropriate assembly strategy.

Table 3. Selected genomic features compared across several vertebrate lineages [19].

Tetrapod taxon	Genome size (Gb)	GC-content	Transposable elements content	
			Range	Mean Value
Mammals	2.2-6.0	~40.9%	33.4%-56.4%	44.5%
Birds	1.2-2.1	~40.2%	4.6%-10.1%	7.8%
Colubroidea	1.5-3.0	39.3-47.8%	33.0%-56.3%	46.2%
Non-colubroid snakes	1.7-2.1	38.8-43.4%	28.7%-48.7%	38.7%
Scincoidea (skinks)	1.3-2.6	43.2-46.1%	34.3%-44.0%	37.6%

3. Understanding snake venom evolution through snake genomes

3.1 Genetic research on snake toxins

Phylogenetics is the cornerstone of our understanding of evolutionary relationships at all taxonomic levels and provides a historical basis for testing and inferring ecological and evolutionary processes

[93–96]. In the past few decades, snake venom and its evolutionary origins have received considerable attention [44,97–99]. Although there is uncertainty and controversy about the origin of the venom system in squamate reptiles, it is generally accepted that the core snake venom system appeared once in the common ancestor of snakes and lizards [97].

Venom is a polygenic trait that has evolved many times in the tree of life, and it serves in both prey capture and defence against predators [98,100]. Unlike many polygenic traits [101,102], venom has a relatively direct pathway from transcription of toxin genes to translation into toxin proteins, which are then stored for use in the venom gland [44,103]. Thus, by combining venom-gland transcriptomics and venom proteomics, we can accurately map the progression from genotype to phenotype in this adaptive trait [98]. Although transcriptome data will vary depending on the environmental conditions the snake was subjected to at the time of collection and/or sampling, as well as on the characteristics of the underlying genotype, transcriptomes represent a sample of the spatiotemporally-expressed genome and can be used as an entry into genome divergence analysis. Genome divergence analysis takes advantage of whole genome and/or transcriptome data to reconstruct phylogenies that chart the relationships among snakes, thus representing a precious resource for studies of snake venom evolution.

3.2 Structural characteristics of the toxin genes in snake genomes

More than 10,000 species of squamate reptiles have evolved over the last 200 million years, making this clade a major component of the vertebrate lineage [104]. The number of protein-coding genes is remarkably constant across vertebrates (including snakes), but vertebrate genomes differ considerably in size, structure, and composition [19]. An important genomic feature in this regard are transposable elements (TEs), which are self-replicating DNA sequences with the ability to insert themselves in new positions in the genome, thereby altering genome structure and gene regulation

[105,106]. Having a high abundance of transposable elements could lead to a very fluid genome where pseudogenization and gene duplication may occur more frequently, thus creating opportunities for neofunctionalization. As such, it is perhaps hardly surprising that TEs are consistently involved in the evolution of snake venom [15,16].

Preliminary research indicates that one of the main differences across snake genomes is the abundance and diversity of TEs, which ranges between 33.0%-56.3% in Colubroidea to 28.7%-48.7% in non-colubroid snakes [18,25,88,91]. For comparison, other reptiles, such as members of the order Scincoidea, have a lower variation in their number of transposable elements (34.3%-44.0%) (Table 3) [19,25,88]. Both abundance and diversity of TEs in snake genomes are exemplified by the genomes of *D. acutus* and *B. jararaca*. The former is made up of 13.84% long interspersed elements (LINEs, e.g. CR1, L1, and L2), 7.96% DNA transposons (e.g. hAT and TcMar elements), and 2.59% retrotransposons (e.g. Gypsy and DIRS elements) [88], whereas the latter comprises 14.6% LINEs with L2/CR1/Rex as the most abundant (8.8% of whole genome). The genomes of *D. acutus* and *O. hannah* have a fairly low-divergence level (<10%) of CR1 and hAT elements from the inferred ancestral consensus sequences, while snakes belonging to more basal-branching clades (e.g. *B. constrictor* and *P. bivittatus*) have more than 20% divergence level [88]. Conversely, CR1 and hAT content is over three times higher in *D. acutus* and *O. hannah* than in *B. constrictor* and *P. bivittatus*, but the latter two species have undergone independent expansion of L2 repeat contents [88]. Another study that highlights genomic differences in TE content in snakes showed that repeat element abundance in the genomes of *D. acutus*, *T. sirtalis*, and *O. hannah* (all part of the Colubroidea clade) were characterized by a higher CR1-like and DNA transposon content compared to the genome of *P. bivittatus* [91]. Overall, repeat elements in the genomes of venomous snakes are generally more active, diverse, and dynamic compared to those of non-venomous species, indicating that different types of transposable

elements may have played multiple important roles in functional regulation of snake genes throughout evolution.

Another TE category that has attracted research attention are microsatellites (short-repeated DNA sequences). Microsatellites are so ubiquitous in certain snake species that a snake genome holds the record for containing the highest microsatellite content in any known eukaryote [19]. Bolstering this claim, a study of 11 viper species found an unprecedented average microsatellite content of 16,214 bp/Mbp [19]. In comparison, the average microsatellite density of four non-venomous snakes was roughly 55% of that amount, i.e. 8,953 bp/Mbp [19]. If this trend holds across a broader selection of species, it could further substantiate the hypothesis that microsatellites have played an important role in snake venom evolution. The same study found that the average genome density of Simple Sequence Repeat (SSR) loci (448-896 loci/Mbp) was roughly twice as large in venomous snake microsatellites as in non-venomous snakes homologs [19]. The study further found that the AATAG loci (which tend to be immediately adjacent to CR1-L3 LINEs in colubroid genomes) in venomous colubroids were increased 75-fold compared to other squamate reptiles and 71-fold compared to non-colubroid snakes [19]. Based on the significant expression of SSRs and LINE-SSR hybrid element content in venomous snakes compared to non-venomous snakes, the study also concluded that SSRs and LINE-SSR hybrid elements may have played key roles in the evolution of snake venom [19]. The dynamics and extent of the influence of SSRs and LINE-SSR on venom evolution therefore represent an intriguing venue for further research.

However, microsatellite content alone cannot explain the course of venom evolution. Indeed, another important factor is the chromosomal location of venom genes. What is known about snake chromosomes is largely based on cytogenetic experimental studies, which have revealed that the majority of snakes have 18 chromosomes (eight macrochromosomes and ten microchromosomes) [18]. It has been observed that a high proportion of venom genes are often located on the long ignored

microchromosomes [13,19], which have higher GC-content and faster recombination rates than macrochromosomes [21]. For example, 37% of all venom genes in the *C. viridis* genome and ca. 57% (27/47 genes) of all annotated venom-related genes in the *P. flavoviridis* genome are located on microchromosomes (Fig. 3) [21,22]. This is the case for *C. tigris* as well, with all genes belonging to the major toxin family in the venom of this species (PLA_2s) located on microchromosome 7 [33]. Phylogenetic analysis of the three most abundant and well-characterized toxin families in *C. viridis* venom (SVMPs, SVSPs, and PLA_2s, all located on microchromosomes) revealed that each toxin gene family represents a distinct set of duplicated genes derived from a single ancestral homolog that produced a monophyletic cluster of venomous paraphyletic lineages [21]. Furthermore, the *C. viridis* genome displays a higher and more variable GC-content and higher gene density on microchromosomes than on macrochromosomes [18]. Therefore, it appears that microchromosomes are enriched with venom genes, which together with their high recombination rate could explain the huge radiation and rapid evolution of venom-related genes [13]. Nonetheless, it should be noted that a substantial percentage of toxin-coding genes are found on macrochromosomes as well. This is evident in *N. naja*, where as many as 16 toxin gene families are located on macrochromosomes [32]. WGS of other venomous snake species will be essential to investigate how and to what extent chromosomal location of genes influences venom evolution.

Interestingly, the chromosome structure of *C. viridis* is comparable to that of *N. naja*. In fact, chromosome 4 of *N. naja* shares syntenic regions with *C. viridis* chromosomes 3 and 5, and chromosomes 5 and 6 of *N. naja* are syntenic with chromosome 5 of *C. viridis* [25]. This might indicate the occurrence of fusion and fission events, respectively [25]. The *N. naja* genome has also been compared to that of *O. hannah* (another elapid, and thus more closely related to *N. naja* than *C. viridis*), where 139 venom gland toxin genes from the *N. naja* genome were cross-referenced with genes in the *O. hannah* genome to find orthologs [25]. The results showed that 96 of the *N. naja* genes had

counterparts in the *O. hannah* genome, while 43 did not [25]. Although some of these 43 genes may be unique to *N. naja*, others may simply not have been annotated in the *O. hannah* genome, possibly due to the high fragmentation of its assembly (which relied on short-reads) [14].

In the future, widespread access to different types of sequencing platforms providing researchers with both short and long reads, complementary tools for genome analysis (Hi-C and CHiCAGO), and higher quality sequence data will likely enable researchers to study snake genomes in greater detail. In turn, this will help elucidate differences and similarities between snake genomes and allow for more fine-grained studies of the structural characteristics of snake venom genes.

3.3 Molecular origin and regulation of snake venom genes

Snake venoms and their evolutionary origins have received substantial attention over the past decades, with more than 15,000 studies published on this topic [13]. Snake venoms have the dual functions of defense against predators and subduction of prey, with predation typically being the primary function [98]. This locks snakes and their prey in an evolutionary arms race, where the prey evolves biological strategies that make it resistant to toxins, and snakes are constantly pressured to optimize and adjust the composition of toxins in their venoms [98]. Indeed, dietary habits have often been indicated as a key driver of adaptive venom evolution in snakes, featuring among the main reasons behind inter- and intraspecific variation in venom composition [107].

Reports on trophic adaptations of snakes are plentiful. As an example, a study showing that venom variation in the Malayan pitviper (*Calloselasma rhodostoma*) throughout its range is significantly associated with the types of prey locally available [108]. This is also the case for the Mangrove catsnake (*Boiga dendrophila*), which was found to possess a 3FTx specific for birds and lizards (the bulk of this snake's diet) but scarcely effective on mammals [109]. However, recent research reported that venom composition in the Mojave rattlesnake (*Crotalus scutulatus*) was

associated with environmental factors (e.g. temperature, seasonality) rather than with diet [110]. This suggests that a more complex scenario of factors could be affecting venom diversity than prey-related drivers alone, as confirmed by the dynamics behind venom variation in the Northern Pacific rattlesnake (*Crotalus oreganus*). In fact, the dichotomy in venom composition observed in this species is consistently influenced not only by coevolution with its prey, but also by genetic distance and elevation-based habitat gradients, in a pattern described as "phenotype matching" of venom characteristics to multiple variables in the snake's native ecosystem [111,112]. The genetic basis underlying such complex adaptive processes could likely provide intriguing insight into the influence of natural selection and phylogenetic relatedness on the evolution of a highly dynamic trait such as snake venom. To this end, whole genome sequencing of snakes will likely be key to conclusively determining the structural and evolutionary features of toxin genes and gene clusters. Analyzing such patterns in a comparative framework would then enable researchers to identify similarities and differences in adaptive drivers of venom evolution at all levels of snake taxonomy and phylogeny.

In recent years, venom evolution has been further explored through genome studies on several species of venomous snakes [13,19,25,88]. One of these studies revealed that the venom gene repertoire of *D. acutus* has a very different composition from those of *O. hannah* and the non-venomous *A. carolinensis* (outgroup), *B. constrictor*, and *P. bivittatus*. These differences are exemplified both by the absence of characteristic venom genes from the *D. acutus* genome relative to the *O. hannah* genome and by the increased gene copy number of other venom gene families, including SVMPs, CTLPs, and SVSPs (Table 1) [88]. Expression of most toxin-encoding genes shared by *D. acutus* and *O. hannah* (especially older genes derived from the last common ancestor of these species) is limited to venom glands or accessory glands [88]. Similarly, newer viper-specific toxin genes are expressed in the venom and accessory glands of *D. acutus*, while equally recent elapid-specific toxin genes are expressed in the venom and accessory glands of *O. hannah* [88]. Interestingly, genes closely related to the elapid-

specific toxin genes expressed in the venom glands of *O. hannah* are expressed in the liver of *D. acutus*, and genes related to viper-specific toxin genes expressed in the venom glands of *D. acutus* are expressed in pooled organs from *O. hannah* [88].

These special expression patterns suggest that these venom genes may originate from metabolic proteins that have undergone subfunctionalization (i.e., paralogs retaining only part of the functional features of the original gene following duplication) or neofunctionalization, as well as that changes in tissue-specific expression have occurred [15,88]. This is in accordance with previous protein-based findings [113,114]. Similarly, analysis of the *O. hannah* genome demonstrated that the regulatory components of the venomous secretion system may have evolved from the pancreas [16]. Several mechanisms likely contribute to the enhanced expression of toxin-coding genes in the venom gland. At the chromosome level, methylation and chromatin accessibility were recently shown to play a prominent role in gene regulation in *C. tigris*. In fact, methylation appears to be significantly more prevalent in non-toxin and unexpressed toxin genes compared to expressed toxin counterparts in the venom gland and pancreas of this species [33]. Furthermore, expressed toxin genes are significantly more accessible chromatin-wise than non-expressed counterparts and non-toxin genes in *C. tigris*, further supporting a joint role for these two factors in toxin gene expression [33]. Another important factor in regulation and expression of toxin genes is the metavenom network, which comprises ~3000 genes that do not code for toxins but actively influence their expression and postgenomic modifications (e.g. protein folding) in the venom gland as housekeeping genes [48]. Interestingly, this network presents highly conserved elements common to even distantly related lineages such as snakes and venomous mammals; on the other hand, snakes (specifically *P. flavoviridis* and *P. mucrosquamatus*) also displayed several unique regulatory genes that were likely co-opted together with neofunctionalized toxin genes absent in other lineages [48].

Gene duplication is thought to be one of the main mechanisms behind venom diversification [115]. The current consensus is that two rounds of whole-genome duplication (2R-WGD) occurred during the evolution of vertebrates [13,116]. A study of the *P. flavoviridis* genome suggests that this resulted in the creation of four paralogs from each of the 18 genes which are ancestral to the toxin and non-toxin genes in *P. flavoviridis* [13]. During the later evolution of venomous snakes, it is likely that one of these four gene copies underwent neo- or subfunctionalization and evolved toxic properties, while the remaining three copies did not [13]. Both the toxin and non-toxin encoding genes subsequently underwent multiplication to different extents (Fig. 4A) [13], as is demonstrated by the multiple gene duplication events detected in the SVMP, SVSP, CTLP, PLA_2, 3FTx, and CRISP gene families in *P. flavoviridis* and *N. naja* [13,25]. The multiplication of toxin and non-toxin genes of *P. flavoviridis* by tandem duplication is also suggested by the retention of two SVMP gene clusters in the same scaffold [13]. The first cluster contains four snake toxin genes and one non-toxin gene, while the second cluster contains three snake toxin genes (Fig. 4B). In comparison, SVSPs are located in three gene clusters, with each cluster containing at least three snake toxin genes (Fig. 4C), while CTLPs are located in two gene clusters containing five and nine CTLP genes, respectively (Fig. 4D) [13]. Molecular phylogenetic analysis of *P. flavoviridis* shows that all toxin genes of a given gene family in this species are homologous to the same toxin gene families found in vipers and elapids, such as *P. mucrosquamatus* (Brown-spotted pitviper) and *O. hannah* [13]. The notion that snake toxin genes massively expanded through gene duplication events and underwent neofunctionalization is also supported by other studies [16,25,117]. For example, the *N. naja* genome assembly contributes to our understanding of the origin of multiple unlinked venom gene clusters and provides new and conclusive evidence that each toxin family stems from a unique set of tandem duplicate genes [25].

Fig 3. *Venom-related gene families in the P. flavoviridis genome.* ***(A)*** *Deduced evolutionary history of venom-related gene families through two rounds of whole-genome duplication (2R-WGD). An original set of 18 genes (shown in the top box) became 72 (four copies each). Then, a single copy of each family was likely co-opted to develop toxic functions, resulting in one snake venom (SV) copy (shown in a pale red box in the right column) and three non-venom (NV) paralogs (shown in the see-through box to the left).* ***(B)*** *Tandem duplications of SVMP genes.* ***(C)*** *Tandem duplications of SVSP genes.* ***(D)*** *Tandem duplications of CTLP genes. Based on Fig. 2 and Fig. S8 from* [13].

While duplication either before or after gene recruitment to the venom gland is an established driving force of venom evolution in snakes, loss of genetic material has been no less pivotal in facilitating diversification of toxin families in certain venomous snake clades. For instance, the interplay between gene duplication and deletion (of entire genes as well as intragenic regions) is remarkable in rattlesnakes (*Crotalus* spp.). These pitvipers present signs of multiple independent losses of ancestral genes coding for SVMPs and neurotoxic PLA_2s – both of which had previously experienced a rampant expansion via repeated duplication episodes – across their phylogenetic tree [37,68]. Intriguingly, different genes underwent deletion among and even within species, such as observed in the Western diamondback rattlesnake (*C. atrox*), the Mojave rattlesnake (*C. scutulatus*), and the Southern Pacific rattlesnake (*C. helleri*) [37,61,68]. This resulted in great haplotype disparity and differential expression of toxin-encoding genes not only between species, but across conspecific individuals as well. Whole genome sequencing of *C. tigris* further corroborated this pattern, as this species is known for its remarkably simple venom composition largely based on neurotoxic PLA_2 isoforms [118]. However, the *C. tigris* genome revealed a deletion of three PLA_2 genes on microchromosome 7 and of ten SVMP genes on microchromosome 1 compared to homologous regions in *C. viridis*, indicating that even such a simple venom phenotype is the result of extensive genomic modifications over evolutionary time [26]. This pattern is not limited to rattlesnakes. For instance, the

Bothrops jararaca genome also displays a great expansion of SVMP genes via duplication upon recruitment in the venom gland, followed by two deletions in the exon 14 region of PII-SVMP genes causing loss of the Cys-rich domain found in PIII-SVMPs [27]. This observation sheds further light on the genomic processes responsible for evolution and differentiation via domain loss in SVMPs, which has occurred in other viper lineages as well [44].

3.4. Adaptive and neutral evolution in snake venom

Determining and unraveling the driving factors behind the dynamic evolutionary processes in snake venom gene families has garnered the interest of scientists for decades – a quest that could only benefit from rising efforts in WGS of venomous snakes. Positive selection appears to be the force behind the evolution of genes involved in predator-prey arms races [119], and it seems to be pervasive across most toxin-related gene families in snakes. Positive selection leaves a well-defined pattern in the genome, with the accumulation of non-synonymous, amino-acid replacing nucleotide substitutions (denoted by KA), over synonymous substitutions (KS) in the gene [120]. In *P. flavoviridis*, the KA/KS ratios of the four main toxin gene families were consistently higher than 1 and/or higher than those reported for non-venom genes (SVMPs: 1.047 ± 0.438, SVSPs: 1.253 ± 0.090, CTLPs: 0.871 ± 0.071, PLA_2s: 1.093 ± 0.062) [13], suggesting positive selection behind the accelerated evolution of the major toxin gene families in this species. Interestingly, *P. flavoviridis* also exhibited KA/KS > 1 in the 3FTx and CRISP gene families, which therefore also displayed a tendency towards accelerated evolution despite being present in far fewer copies [13]. Similarly, a high KA/KS ratio (2.034 ± 0.818) was observed for the 3FTx gene family in the *N. naja* genome, again pointing towards rapid differentiation and functional diversification for these genes [25]. Conversely, when KA/KS < 1 is indicative of either neutral selection (random substitutions that confer neither evolutionary advantages nor disadvantages)

or purifying selection (i.e. removal of mutations that usually tend to be deleterious as they appear in conserved areas of the gene). In the *P. flavoviridis* genome study, all non-dominant toxin gene families had a KA/KS < 1 (Mean ± SE = 0.512 ± 0.018), indicating a more neutral nucleotide substitution and the maintenance of similarity between gene copies [13].

New -omics tools and methods are rapidly advancing our knowledge of the mechanisms behind venom evolution [121]. In particular, WGS has introduced advantages to snake venom research, as WGS data can be used to identify structural variants, including inversions (Fig. 3A-B), insertions (Fig. 3C), deletions, tandem repeats (Fig. 3A-C), transposable elements (TEs), and other repeat content [19,122]. An increasing number of studies report venom variation at different levels, such as ontogenetic, within-species, and between-species [44,120,123–126]. Once the reference genome of a species is available, population genomics can contribute to the identification of such intra- or interspecific variation. This further enhances the study of venom regulation, helping understand the evolution of complex regulatory networks [26]. Although it is generally acknowledged that positive selection appears to be the main driver behind venom evolution, genomic tools allow zooming in on specific venom-related genes to infer the role of neutral evolutionary processes, i.e. genetic drift or random changes in allele frequencies [127]. Genetic drift contributes to the accumulation of random neutral variation, which serves as the basis for natural selection to act upon in response to new evolutionary pressures [128]. Although most research to date has focused on the adaptive processes explaining venom evolution, recent studies have started assessing the role of such neutral forces in shaping venom characteristics. For example, genetic drift was identified as a prominent factor behind sequence divergence in venom genes in *P. mucrosquamatus*, where dominant toxin-encoding genes displayed relaxed selective constraints for deleterious mutations despite statistically significant rates of positive selection [22]. Furthermore, it has been shown that variation in expression of the myotoxin, crotamine, in the Eastern diamondback rattlesnake (*Crotalus adamanteus*) and the South American

rattlesnake (*Crotalus durissus*) is significantly more correlated with differences in number of duplication-derived gene copies between populations than with adaptive divergence in the sequences themselves [129,130].

Fig 4. *Syntenic comparison of toxin gene clusters. Comparison showing the 3FTx, CRISP, and SVMP genes in N. naja, and C. viridis genomes. Orthologous gene pairs are indicated by the line linked across the corresponding genomic regions. Based on Fig. 4 and Extended Fig. 4 from* [32].

The strength at which genetic drift acts on the genome is inversely proportional to effective population size (N_e, namely the number of reproductive individuals that actually produce offspring) [128]. N_e greatly contributes to sequence variation, as the fate of a favourable mutation spreading is controlled by N_e and the strength of selection [131,132]. A prime example of this pattern in snake venom evolution is presented by the Eastern massasauga rattlesnake (*Sistrurus catenatus*), a threatened species whose range consists of several scattered populations largely isolated from each other. Although the influence of genetic drift on venom evolution in this species is currently weak, it is likely to increase dramatically over time once the impact of drift is augmented due to the low N_e found in most populations [133]. Thus, complete genomes obtained through WGS together with cDNA libraries can expand our knowledge of the effects of selection on venom genes, with great potential to either corroborate or challenge the current positive selection-centered view of snake venom evolution.

4. Conclusions and perspectives

WGS is a revolutionary advance in genetic research that has only recently been applied to the fields of herpetology and toxinology. Nonetheless, sequencing of complete snake genomes has already shed

light on the evolutionary history of toxin-encoding genes as well as their expression patterns in the venom gland. In the future, WGS may be harnessed to obtain a better understanding of the molecular mechanisms involved in snake evolution [4,97], find new bioactive molecules with potential clinical applications, and provide valuable information for antivenom development [33]. As only 21 complete snake genomes are currently available, there is ample opportunity for genomic research on the remaining thousands of snake species, including medically relevant venomous representatives. With the increasing power of sequencing technologies, the field of snake genomics is indeed likely to expand significantly in the years to come, with multiple complete genomes already in the process of being sequenced or published. However, this will not come without challenges, as the interplay of dietary and environmental factors that has fueled venom diversification via gene duplication, recruitment, and neofunctionalization events makes it difficult to assemble whole venomous snake genomes. Another factor adding to the complexity of *de novo* genome assembly is the high content of repeat sequences in snake genomes. Some of these challenges might be adequately addressed by utilizing third-generation sequencing technology. As the costs and error rates of this and other approaches decrease, they are certain to be used more widely in snake genome research. In turn, the assembly of more venomous snake genomes will allow us to explore adaptation and venom evolution at all phylogenetic levels, bringing a new perspective to the study of snake genomes and venoms.

Data availability

Not applicable.

Competing interests

WR, WZ, and SL are employees at the BGI.

Funding

This research was supported by the Beijing Genomics Institute and the Technical University of Denmark. MEA is funded by the Independent Research Fund Denmark (7027-00147B). CK is funded by Innovation Fund Denmark (9065-00007B). TPJ is funded under Marie Sklodowska-Curie grant agreement no. 713683 (COFUNDfellowsDTU).

Authors' contributions

AHL and SL conceived the project. AHL, WR, KK, TPJ, CK, CTW, WZ, SG, LS, MMD, BJM and MEA structured the draft and provided final editing. AHL, KK, TPJ, and WR coordinated and drafted the manuscript and implemented comments provided by all authors. All authors contributed critically to the scientific content. All authors read and approved the final manuscript.

References

1. Burbrink FT, Pyron RA. The Taming of the Skew: Estimating Proper Confidence Intervals for Divergence Dates. Collins T, editor. *Systematic Biology*. 2008; doi: 10.1080/10635150802040605.
2. Wallach V, Williams KL, Boundy J. Snakes of the World: A Catalogue of Living and Extinct Species. 1st edition. Boca Raton: CRC Press;
3. Da Silva FO, Fabre A-C, Savriama Y, Ollonen J, Mahlow K, Herrel A, et al.. The ecological origins of snakes as revealed by skull evolution. *Nat Commun*. 2018; doi: 10.1038/s41467-017-02788-3.
4. Vonk FJ, Admiraal JF, Jackson K, Reshef R, de Bakker MAG, Vanderschoot K, et al.. Evolutionary origin and development of snake fangs. *Nature*. 2008; doi: 10.1038/nature07178.
5. Saviola AJ, Chiszar D, Busch C, Mackessy SP. Molecular basis for prey relocation in viperid snakes. *BMC Biology*. 2013; doi: 10.1186/1741-7007-11-20.
6. Gracheva EO, Ingolia NT, Kelly YM, Cordero-Morales JF, Hollopeter G, Chesler AT, et al.. Molecular basis of infrared detection by snakes. *Nature*. 2010; doi: 10.1038/nature08943.

7. Castoe TA, de Koning APJ, Hall KT, Card DC, Schield DR, Fujita MK, et al.. The Burmese python genome reveals the molecular basis for extreme adaptation in snakes. *Proceedings of the National Academy of Sciences*. 2013; doi: 10.1073/pnas.1314475110.
8. Greene HW, Fogden M, Fogden P. Snakes: The Evolution of Mystery in Nature. First edition. Oxford: University of California Press;
9. Cohn MJ, Tickle C. Developmental basis of limblessness and axial patterning in snakes. *Nature*. 1999; doi: 10.1038/20944.
10. Di-Poï N, Montoya-Burgos JI, Miller H, Pourquié O, Milinkovitch MC, Duboule D. Changes in *Hox* genes' structure and function during the evolution of the squamate body plan. *Nature*. 2010; doi: 10.1038/nature08789.
11. Guerreiro I, Nunes A, Woltering JM, Casaca A, Novoa A, Vinagre T, et al.. Role of a polymorphism in a Hox/Pax-responsive enhancer in the evolution of the vertebrate spine. *Proceedings of the National Academy of Sciences*. 2013; doi: 10.1073/pnas.1300592110.
12. Vicoso B, Emerson JJ, Zektser Y, Mahajan S, Bachtrog D. Comparative Sex Chromosome Genomics in Snakes: Differentiation, Evolutionary Strata, and Lack of Global Dosage Compensation. *PLOS Biology*. 2013; doi: 10.1371/journal.pbio.1001643.
13. Shibata H, Chijiwa T, Oda-Ueda N, Nakamura H, Yamaguchi K, Hattori S, et al.. The habu genome reveals accelerated evolution of venom protein genes. *Scientific Reports*. 2018; doi: 10.1038/s41598-018-28749-4.
14. Uetz, P., Freed, P, Hošek, J: The Reptile Database. http://www.reptile-database.org/ Accessed 2020 Jun 8.
15. Kerkkamp H, Kini R, Pospelov A, Vonk F, Henkel C, Richardson M. Snake Genome Sequencing: Results and Future Prospects. *Toxins*. 2016; doi: 10.3390/toxins8120360.
16. Vonk FJ, Casewell NR, Henkel CV, Heimberg AM, Jansen HJ, McCleary RJR, et al.. The king cobra genome reveals dynamic gene evolution and adaptation in the snake venom system. *Proceedings of the National Academy of Sciences*. 2013; doi: 10.1073/pnas.1314702110.
17. Bradnam KR, Fass JN, Alexandrov A, Baranay P, Bechner M, Birol I, et al.. Assemblathon 2: evaluating de novo methods of genome assembly in three vertebrate species. *GigaScience*. 2013; doi: 10.1186/2047-217X-2-10.
18. Schield DR, Card DC, Hales NR, Perry BW, Pasquesi GM, Blackmon H, et al.. The origins and evolution of chromosomes, dosage compensation, and mechanisms underlying venom regulation in snakes. *Genome Res*. 2019; doi: 10.1101/gr.240952.118.
19. Pasquesi GIM, Adams RH, Card DC, Schield DR, Corbin AB, Perry BW, et al.. Squamate reptiles challenge paradigms of genomic repeat element evolution set by birds and mammals. *Nat Commun*. 2018; doi: 10.1038/s41467-018-05279-1.
20. Ullate-Agote A, Milinkovitch MC, Tzika AC. The genome sequence of the corn snake (Pantherophis guttatus), a valuable resource for EvoDevo studies in squamates. *The International Journal of Developmental Biology*. 2014; doi: 10.1387/ijdb.150060at.
21. Li J-T, Gao Y-D, Xie L, Deng C, Shi P, Guan M-L, et al.. Comparative genomic investigation of high-elevation adaptation in ectothermic snakes. *Proceedings of the National Academy of Sciences*. 2018; doi: 10.1073/pnas.1805348115.
22. Aird SD, Arora J, Barua A, Qiu L, Terada K, Mikheyev AS. Population Genomic Analysis of a Pitviper Reveals Microevolutionary Forces Underlying Venom Chemistry. *Genome Biol Evol*. 2017; doi: 10.1093/gbe/evx199.
23. : Vipera berus berus isolate:VBER.BE-female (ID 170536) - BioProject - NCBI. https://www.ncbi.nlm.nih.gov/bioproject/PRJNA170536 Accessed 2019 Nov 27.

24. Gilbert C, Meik JM, Dashevsky D, Card DC, Castoe TA, Schaack S. Endogenous hepadnaviruses, bornaviruses and circoviruses in snakes. *Proc Biol Sci*. 2014; doi: 10.1098/rspb.2014.1122.

25. Suryamohan K, Krishnankutty SP, Guillory J, Jevit M, Schröder MS, Wu M, et al.. The Indian cobra reference genome and transcriptome enables comprehensive identification of venom toxins. *Nature Genetics*. Nature Publishing Group; 2020; doi: 10.1038/s41588-019-0559-8.

26. Margres MJ, Rautsaw RM, Strickland JL, Mason AJ, Schramer TD, Hofmann EP, et al.. The Tiger Rattlesnake genome reveals a complex genotype underlying a simple venom phenotype. *PNAS*. National Academy of Sciences; 2021; doi: 10.1073/pnas.2014634118.

27. Almeida DD, Viala VL, Nachtigall PG, Broe M, Gibbs HL, Serrano SM de T, et al.. Tracking the recruitment and evolution of snake toxins using the evolutionary context provided by the Bothrops jararaca genome. *PNAS*. National Academy of Sciences; 2021; doi: 10.1073/pnas.2015159118.

28. Peng C, Ren J-L, Deng C, Jiang D, Wang J, Qu J, et al.. The genome of Shaw's sea snake (Hydrophis curtus) reveals secondary adaptation to its marine environment. *Mol Biol Evol*. doi: 10.1093/molbev/msaa043.

29. Ochoa A, Gibbs HL. Genomic signatures of inbreeding and mutation load in a threatened rattlesnake. *Molecular Ecology*. doi: 10.1111/mec.16147.

30. Vonk FJ, Jackson K, Doley R, Madaras F, Mirtschin PJ, Vidal N. Snake venom: From fieldwork to the clinic: Recent insights into snake biology, together with new technology allowing high-throughput screening of venom, bring new hope for drug discovery. *BioEssays*. 2011; doi: 10.1002/bies.201000117.

31. Windley MJ, Herzig V, Dziemborowicz SA, Hardy MC, King GF, Nicholson GM. Spider-Venom Peptides as Bioinsecticides. *Toxins*. 2012; doi: 10.3390/toxins4030191.

32. Hucho F. Toxins as Tools in Neurochemistry. *Angewandte Chemie International Edition in English*. 1995; doi: 10.1002/anie.199500391.

33. Laustsen AH. Guiding recombinant antivenom development by omics technologies. *New Biotechnology*. 2018; doi: 10.1016/j.nbt.2017.05.005.

34. Majoros WH, Pertea M, Salzberg SL. TigrScan and GlimmerHMM: two open source ab initio eukaryotic gene-finders. *Bioinformatics*. 2004; doi: 10.1093/bioinformatics/bth315.

35. Liu X, Zheng Q, Vrettos N, Maragkakis M, Alexiou P, Gregory BD, et al.. A MicroRNA Precursor Surveillance System in Quality Control of MicroRNA Synthesis. *Mol Cell*. 2014; doi: 10.1016/j.molcel.2014.07.017.

36. Collins JE, White S, Searle SMJ, Stemple DL. Incorporating RNA-seq data into the Zebrafish Ensembl Gene Build. *Genome Res*. 2012; doi: 10.1101/gr.137901.112.

37. Dowell NL, Giorgianni MW, Kassner VA, Selegue JE, Sanchez EE, Carroll SB. The Deep Origin and Recent Loss of Venom Toxin Genes in Rattlesnakes. *Current Biology*. 2016; doi: 10.1016/j.cub.2016.07.038.

38. Tekaia F. Inferring Orthologs: Open Questions and Perspectives. *Genomics Insights*. 2016; doi: 10.4137/GEI.S37925.

39. Viala VL, Hildebrand D, Trusch M, Fucase TM, Sciani JM, Pimenta DC, et al.. Venomics of the Australian eastern brown snake (Pseudonaja textilis): Detection of new venom proteins and splicing variants. *Toxicon*. 2015; doi: 10.1016/j.toxicon.2015.06.005.

40. Ogawa T, Oda-Ueda N, Hisata K, Nakamura H, Chijiwa T, Hattori S, et al.. Alternative mRNA Splicing in Three Venom Families Underlying a Possible Production of Divergent Venom Proteins of the Habu Snake, Protobothrops flavoviridis. *Toxins (Basel)*. 2019; doi: 10.3390/toxins11100581.

41. Siigur E, Aaspõllu A, Siigur J. Sequence diversity of Vipera lebetina snake venom gland serine proteinase homologs – result of alternative-splicing or genome alteration. *Gene*. 2001; doi: 10.1016/S0378-1119(00)00571-0.

42. Sunagar K, Khochare S, Senji Laxme RR, Attarde S, Dam P, Suranse V, et al.. A Wolf in Another Wolf's Clothing: Post-Genomic Regulation Dictates Venom Profiles of Medically-Important Cryptic Kraits in India. *Toxins*. Multidisciplinary Digital Publishing Institute; 2021; doi: 10.3390/toxins13010069.

43. Rokyta DR, Margres MJ, Calvin K. Post-transcriptional Mechanisms Contribute Little to Phenotypic Variation in Snake Venoms. *G3 (Bethesda)*. 2015; doi: 10.1534/g3.115.020578.

44. Casewell NR, Wagstaff SC, Wuster W, Cook DAN, Bolton FMS, King SI, et al.. Medically important differences in snake venom composition are dictated by distinct postgenomic mechanisms. *Proceedings of the National Academy of Sciences*. 2014; doi: 10.1073/pnas.1405484111.

45. Barua A, Mikheyev AS. An ancient, conserved gene regulatory network led to the rise of oral venom systems. *Proc Natl Acad Sci U S A*. 2021; doi: 10.1073/pnas.2021311118.

46. Elshire RJ, Glaubitz JC, Sun Q, Poland JA, Kawamoto K, Buckler ES, et al.. A Robust, Simple Genotyping-by-Sequencing (GBS) Approach for High Diversity Species. *PLOS ONE*. Public Library of Science; 2011; doi: 10.1371/journal.pone.0019379.

47. Cariou M, Duret L, Charlat S. Is RAD-seq suitable for phylogenetic inference? An in silico assessment and optimization. *Ecology and Evolution*. 2013; doi: 10.1002/ece3.512.

48. Ellegren H. Genome sequencing and population genomics in non-model organisms. *Trends in Ecology & Evolution*. Elsevier; 2014; doi: 10.1016/j.tree.2013.09.008.

49. Laustsen A, Engmark M, Milbo C, Johannesen J, Lomonte B, Gutiérrez J, et al.. From Fangs to Pharmacology: The Future of Snakebite Envenoming Therapy. *Current Pharmaceutical Design*. 2016; doi: 10.2174/1381612822666160623073438.

50. Tasoulis T, Isbister GK. A Review and Database of Snake Venom Proteomes. *Toxins*. 2017; doi: 10.3390/toxins9090290.

51. Kini RM, Doley R. Structure, function and evolution of three-finger toxins: mini proteins with multiple targets. *Toxicon*. 2010; doi: 10.1016/j.toxicon.2010.07.010.

52. Fry BG, Wüster W, Kini RM, Brusic V, Khan A, Venkataraman D, et al.. Molecular Evolution and Phylogeny of Elapid Snake Venom Three-Finger Toxins. *J Mol Evol*. 2003; doi: 10.1007/s00239-003-2461-2.

53. Nirthanan S. Snake three-finger α-neurotoxins and nicotinic acetylcholine receptors: molecules, mechanisms and medicine. *Biochemical Pharmacology*. 2020; doi: 10.1016/j.bcp.2020.114168.

54. Gasanov SE, Dagda RK, Rael ED. Snake Venom Cytotoxins, Phospholipase A2s, and Zn2+-dependent Metalloproteinases: Mechanisms of Action and Pharmacological Relevance. *J Clin Toxicol*. 4:10001812014;

55. Lomonte B, Gutiérrez JM. Phospholipases A2 from viperidae snake venoms: how do they induce skeletal muscle damage? *Acta Chim Slov*. 58:647–592011;

56. Gutiérrez JM, Calvete JJ, Habib AG, Harrison RA, Williams DJ, Warrell DA. Snakebite envenoming. *Nature Reviews Disease Primers*. 2017; doi: 10.1038/nrdp.2017.63.

57. Tsai I-H. Snake Venom Phospholipase A2: Evolution and Diversity. In: Gopalakrishnakone P, Calvete JJ, editors. *Venom Genomics and Proteomics*. Dordrecht: Springer Netherlands; p. 291–306.

58. Kordiš D. Evolution of phospholipase A2 toxins in venomous animals. *Acta Chim Slov*. 58:638–462011;

59. Manjunatha Kini R. Excitement ahead: structure, function and mechanism of snake venom phospholipase A2 enzymes. *Toxicon*. 2003; doi: 10.1016/j.toxicon.2003.11.002.

60. Ohno M, Chijiwa T, Oda-Ueda N, Ogawa T, Hattori S. Molecular evolution of myotoxic phospholipases A2 from snake venom. *Toxicon*. 2003; doi: 10.1016/j.toxicon.2003.11.003.

61. Dowell NL, Giorgianni MW, Griffin S, Kassner VA, Selegue JE, Sanchez EE, et al.. Extremely Divergent Haplotypes in Two Toxin Gene Complexes Encode Alternative Venom Types within Rattlesnake Species. *Current Biology*. 2018; doi: 10.1016/j.cub.2018.02.031.

62. Markland FS, Swenson S. Snake venom metalloproteinases. *Toxicon*. 2013; doi: 10.1016/j.toxicon.2012.09.004.
63. Gutiérrez JM, Rucavado A. Snake venom metalloproteinases: their role in the pathogenesis of local tissue damage. *Biochimie*. 82:841–502000;
64. Gutiérrez JM, Escalante T, Rucavado A, Herrera C, Fox JW. A Comprehensive View of the Structural and Functional Alterations of Extracellular Matrix by Snake Venom Metalloproteinases (SVMPs): Novel Perspectives on the Pathophysiology of Envenoming. *Toxins*. 2016; doi: 10.3390/toxins8100304.
65. Sanchez EF, Flores-Ortiz RJ, Alvarenga VG, Eble JA. Direct Fibrinolytic Snake Venom Metalloproteinases Affecting Hemostasis: Structural, Biochemical Features and Therapeutic Potential. *Toxins (Basel)*. 2017; doi: 10.3390/toxins9120392.
66. Kini R, Koh C. Metalloproteases Affecting Blood Coagulation, Fibrinolysis and Platelet Aggregation from Snake Venoms: Definition and Nomenclature of Interaction Sites. *Toxins*. 2016; doi: 10.3390/toxins8100284.
67. Casewell NR, Wagstaff SC, Harrison RA, Renjifo C, Wüster W. Domain Loss Facilitates Accelerated Evolution and Neofunctionalization of Duplicate Snake Venom Metalloproteinase Toxin Genes. *Mol Biol Evol*. Oxford Academic; 2011; doi: 10.1093/molbev/msr091.
68. Giorgianni MW, Dowell NL, Griffin S, Kassner VA, Selegue JE, Carroll SB. The origin and diversification of a novel protein family in venomous snakes. *Proc Natl Acad Sci USA*. 2020; doi: 10.1073/pnas.1920011117.
69. Brust A, Sunagar K, Undheim EAB, Vetter I, Yang DC, Casewell NR, et al.. Differential Evolution and Neofunctionalization of Snake Venom Metalloprotease Domains *. *Molecular & Cellular Proteomics*. Elsevier; 2013; doi: 10.1074/mcp.M112.023135.
70. Moura-da-Silva A, Almeida M, Portes-Junior J, Nicolau C, Gomes-Neto F, Valente R. Processing of Snake Venom Metalloproteinases: Generation of Toxin Diversity and Enzyme Inactivation. *Toxins*. 2016; doi: 10.3390/toxins8060183.
71. Serrano SMT. The long road of research on snake venom serine proteinases. *Toxicon*. 2013; doi: 10.1016/j.toxicon.2012.09.003.
72. Kunalan S, Othman I, Syed Hassan S, Hodgson WC. Proteomic Characterization of Two Medically Important Malaysian Snake Venoms, Calloselasma rhodostoma (Malayan Pit Viper) and Ophiophagus hannah (King Cobra). *Toxins*. 2018; doi: 10.3390/toxins10110434.
73. Kang TS, Georgieva D, Genov N, Murakami MT, Sinha M, Kumar RP, et al.. Enzymatic toxins from snake venom: structural characterization and mechanism of catalysis. *The FEBS Journal*. 2011; doi: 10.1111/j.1742-4658.2011.08115.x.
74. White J. Snake venoms and coagulopathy. *Toxicon*. 2005; doi: 10.1016/j.toxicon.2005.02.030.
75. Kini RM. The intriguing world of prothrombin activators from snake venom. *Toxicon*. 2005; doi: 10.1016/j.toxicon.2005.02.019.
76. Markland FS. Snake venoms and the hemostatic system. *Toxicon*. 1998; doi: 10.1016/S0041-0101(98)00126-3.
77. Meenakshisundaram R, Sweni S, Thirumalaikolundusubramanian P. Hypothesis of snake and insect venoms against Human Immunodeficiency Virus: a review. *AIDS Res Ther*. 2009; doi: 10.1186/1742-6405-6-25.
78. Delsuc F, Brinkmann H, Philippe H. Phylogenomics and the reconstruction of the tree of life. *Nat Rev Genet*. 2005; doi: 10.1038/nrg1603.
79. Hall JB, Cobb VA, Cahoon AB. The complete mitochondrial DNA sequence of *Crotalus horridus* (timber rattlesnake). *Mitochondrial DNA*. 2013; doi: 10.3109/19401736.2012.722999.

80. St Pierre L, Masci PP, Filippovich I, Sorokina N, Marsh N, Miller DJ, et al.. Comparative analysis of prothrombin activators from the venom of Australian elapids. *Mol Biol Evol*. 2005; doi: 10.1093/molbev/msi181.

81. Earl STH, Birrell GW, Wallis TP, St Pierre LD, Masci PP, de Jersey J, et al.. Post-translational modification accounts for the presence of varied forms of nerve growth factor in Australian elapid snake venoms. *Proteomics*. 2006; doi: 10.1002/pmic.200600263.

82. McGlothlin JW, Chuckalovcak JP, Janes DE, Edwards SV, Feldman CR, Brodie ED, et al.. Parallel Evolution of Tetrodotoxin Resistance in Three Voltage-Gated Sodium Channel Genes in the Garter Snake Thamnophis sirtalis. *Mol Biol Evol*. 2014; doi: 10.1093/molbev/msu237.

83. Schield DR, Perry BW, Pasquesi GIM, Orton RW, Nikolakis ZL, Westfall AK, et al.. Applications of Genomics and Related Technologies for Studying Reptile Venoms. *Handbook of Venoms and Toxins of Reptiles*. 2nd ed. CRC Press;

84. . Finishing the euchromatic sequence of the human genome. *Nature*. 2004; doi: 10.1038/nature03001.

85. Seppey M, Manni M, Zdobnov EM. BUSCO: Assessing Genome Assembly and Annotation Completeness. In: Kollmar M, editor. *Gene Prediction: Methods and Protocols*. New York, NY: Springer;

86. Jauhal AA, Newcomb RD. Assessing genome assembly quality prior to downstream analysis: N50 versus BUSCO. *Molecular Ecology Resources*. 2021; doi: 10.1111/1755-0998.13364.

87. Edwards R, Amos T, Tang J, Cawood B, Rispin S, Tuipulotu DE, et al.. <p>Pseudodiploid pseudo-long-read whole genome sequencing and assembly of *Pseudonaja textilis* (eastern brown snake) and *Notechis scutatus* (mainland tiger snake)</p>. *F1000Research*. 2018; doi: 10.7490/f1000research.1115550.1.

88. Yin W, Wang Z, Li Q, Lian J, Zhou Y, Lu B, et al.. Evolutionary trajectories of snake genes and genomes revealed by comparative analyses of five-pacer viper. *Nat Commun*. 2016; doi: 10.1038/ncomms13107.

89. Le TS, Yang F-J, Lo Y-H, Chang TC, Hsu J-C, Kao C-Y, et al.. Non-Mendelian assortment of homologous autosomes of different sizes in males is the ancestral state in the Caenorhabditis lineage. *Scientific Reports*. Nature Publishing Group; 2017; doi: 10.1038/s41598-017-13215-4.

90. Birdsell JA. Integrating Genomics, Bioinformatics, and Classical Genetics to Study the Effects of Recombination on Genome Evolution. *Mol Biol Evol*. 2002; doi: 10.1093/oxfordjournals.molbev.a004176.

91. Perry BW, Card DC, McGlothlin JW, Pasquesi GIM, Adams RH, Schield DR, et al.. Molecular Adaptations for Sensing and Securing Prey and Insight into Amniote Genome Diversity from the Garter Snake Genome. *Genome Biol Evol*. 2018; doi: 10.1093/gbe/evy157.

92. Benjamini Y, Speed TP. Summarizing and correcting the GC content bias in high-throughput sequencing. *Nucleic Acids Res*. 2012; doi: 10.1093/nar/gks001.

93. Figueroa A, McKelvy AD, Grismer LL, Bell CD, Lailvaux SP. A Species-Level Phylogeny of Extant Snakes with Description of a New Colubrid Subfamily and Genus. *PLOS ONE*. 2016; doi: 10.1371/journal.pone.0161070.

94. Harvey PH, Harvey PP, Harvey R in BD of ZPH, Pagel MD, Pagel MD. The Comparative Method in Evolutionary Biology. Oxford University Press;

95. Whelan S, Liò P, Goldman N. Molecular phylogenetics: state-of-the-art methods for looking into the past. *Trends Genet*. 2001; doi: 10.1016/s0168-9525(01)02272-7.

96. : Phylogenetic Methods Come of Age: Testing Hypotheses in an Evolutionary Context | Science. https://science.sciencemag.org/content/276/5310/227 Accessed 2021 Mar 21.

97. Fry BG, Vidal N, Norman JA, Vonk FJ, Scheib H, Ramjan SFR, et al.. Early evolution of the venom system in lizards and snakes. *Nature*. 2006; doi: 10.1038/nature04328.

98. Casewell NR, Wüster W, Vonk FJ, Harrison RA, Fry BG. Complex cocktails: the evolutionary novelty of venoms. *Trends in Ecology & Evolution*. 2013; doi: 10.1016/j.tree.2012.10.020.
99. Fry BG, Scheib H, Weerd L van der, Young B, McNaughtan J, Ramjan SFR, et al.. Evolution of an Arsenal: Structural and Functional Diversification of the Venom System in the Advanced Snakes (Caenophidia). *Molecular & Cellular Proteomics*. 2008; doi: 10.1074/mcp.M700094-MCP200.
100. Gibbs HL, Mackessy SP. Functional basis of a molecular adaptation: Prey-specific toxic effects of venom from Sistrurus rattlesnakes. *Toxicon*. 2009; doi: 10.1016/j.toxicon.2009.01.034.
101. : Comparative studies of gene expression and the evolution of gene regulation | Nature Reviews Genetics. https://www.nature.com/articles/nrg3229 Accessed 2021 Mar 21.
102. Shapiro MD, Marks ME, Peichel CL, Blackman BK, Nereng KS, Jónsson B, et al.. Genetic and developmental basis of evolutionary pelvic reduction in threespine sticklebacks. *Nature*. 2004; doi: 10.1038/nature02415.
103. Margres MJ, McGivern JJ, Wray KP, Seavy M, Calvin K, Rokyta DR. Linking the transcriptome and proteome to characterize the venom of the eastern diamondback rattlesnake (Crotalus adamanteus). *J Proteomics*. 2014; doi: 10.1016/j.jprot.2013.11.001.
104. Zheng Y, Wiens JJ. Combining phylogenomic and supermatrix approaches, and a time-calibrated phylogeny for squamate reptiles (lizards and snakes) based on 52 genes and 4162 species. *Molecular Phylogenetics and Evolution*. 2016; doi: 10.1016/j.ympev.2015.10.009.
105. Feiner N. Accumulation of transposable elements in Hox gene clusters during adaptive radiation of Anolis lizards. *Proceedings of the Royal Society B: Biological Sciences*. 2016; doi: 10.1098/rspb.2016.1555.
106. Platt RN, Vandewege MW, Ray DA. Mammalian transposable elements and their impacts on genome evolution. *Chromosome Res*. 2018; doi: 10.1007/s10577-017-9570-z.
107. Barlow A, Pook CE, Harrison RA, Wüster W. Coevolution of diet and prey-specific venom activity supports the role of selection in snake venom evolution. *Proceedings of the Royal Society B: Biological Sciences*. 2009; doi: 10.1098/rspb.2009.0048.
108. Daltry JC, Wüster W, Thorpe RS. Diet and snake venom evolution. *Nature*. 1996; doi: 10.1038/379537a0.
109. Pawlak J, Mackessy SP, Fry BG, Bhatia M, Mourier G, Fruchart-Gaillard C, et al.. Denmotoxin, a Three-finger Toxin from the Colubrid Snake Boiga dendrophila (Mangrove Catsnake) with Bird-specific Activity. *J Biol Chem*. American Society for Biochemistry and Molecular Biology; 2006; doi: 10.1074/jbc.M605850200.
110. Zancolli G, Calvete JJ, Cardwell MD, Greene HW, Hayes WK, Hegarty MJ, et al.. When one phenotype is not enough: divergent evolutionary trajectories govern venom variation in a widespread rattlesnake species. *Proceedings of the Royal Society B*. The Royal Society; 2019; doi: 10.1098/rspb.2018.2735.
111. Holding ML, Biardi JE, Gibbs HL. Coevolution of venom function and venom resistance in a rattlesnake predator and its squirrel prey. *Proceedings of the Royal Society B: Biological Sciences*. Royal Society; 2016; doi: 10.1098/rspb.2015.2841.
112. Holding ML, Margres MJ, Rokyta DR, Gibbs HL. Local prey community composition and genetic distance predict venom divergence among populations of the northern Pacific rattlesnake (Crotalus oreganus). *Journal of Evolutionary Biology*. 2018; doi: 10.1111/jeb.13347.
113. Fry BG. From genome to “venome”: Molecular origin and evolution of the snake venom proteome inferred from phylogenetic analysis of toxin sequences and related body proteins. *Genome Research*. 2005; doi: 10.1101/gr.3228405.
114. Fry BG, Roelants K, Champagne DE, Scheib H, Tyndall JDA, King GF, et al.. The Toxicogenomic Multiverse: Convergent Recruitment of Proteins Into Animal Venoms. *Annual Review of Genomics and Human Genetics*. 2009; doi: 10.1146/annurev.genom.9.081307.164356.

115. Conant GC, Wolfe KH. Turning a hobby into a job: How duplicated genes find new functions. *Nature Reviews Genetics*. 2008; doi: 10.1038/nrg2482.
116. Holland PW, Garcia-Fernàndez J, Williams NA, Sidow A. Gene duplications and the origins of vertebrate development. *Dev Suppl*. :125–33 1994;
117. Casewell NR, Huttley GA, Wüster W. Dynamic evolution of venom proteins in squamate reptiles. *Nature Communications*. Nature Publishing Group; 2012; doi: 10.1038/ncomms2065.
118. Calvete JJ, Pérez A, Lomonte B, Sánchez EE, Sanz L. Snake Venomics of Crotalus tigris: The Minimalist Toxin Arsenal of the Deadliest Neartic Rattlesnake Venom. Evolutionary Clues for Generating a Pan-Specific Antivenom against Crotalid Type II Venoms. *J Proteome Res*. American Chemical Society; 2012; doi: 10.1021/pr201021d.
119. Charlesworth B, Charlesworth D. Elements of evolutionary genetics, 1st edition | macmillan learning for instructors.
120. Casewell NR, Jackson TNW, Laustsen AH, Sunagar K. Causes and Consequences of Snake Venom Variation. *Trends in Pharmacological Sciences*. 2020; doi: 10.1016/j.tips.2020.05.006.
121. Reyes-Velasco J, Card DC, Andrew AL, Shaney KJ, Adams RH, Schield DR, et al.. Expression of Venom Gene Homologs in Diverse Python Tissues Suggests a New Model for the Evolution of Snake Venom. *Molecular Biology and Evolution*. 2015; doi: 10.1093/molbev/msu294.
122. Tattini L, D'Aurizio R, Magi A. Detection of Genomic Structural Variants from Next-Generation Sequencing Data. *Front Bioeng Biotechnol*. 2015; doi: 10.3389/fbioe.2015.00092.
123. Massey DJ, Calvete JJ, Sánchez EE, Sanz L, Richards K, Curtis R, et al.. Venom variability and envenoming severity outcomes of the Crotalus scutulatus scutulatus (Mojave rattlesnake) from Southern Arizona. *Journal of Proteomics*. 2012; doi: 10.1016/j.jprot.2012.02.035.
124. Pla D, Sanz L, Quesada-Bernat S, Villalta M, Baal J, Chowdhury MAW, et al.. Phylovenomics of Daboia russelii across the Indian subcontinent. Bioactivities and comparative in vivo neutralization and in vitro third-generation antivenomics of antivenoms against venoms from India, Bangladesh and Sri Lanka. *Journal of Proteomics*. 2019; doi: 10.1016/j.jprot.2019.103443.
125. Durban J, Sanz L, Trevisan-Silva D, Neri-Castro E, Alagón A, Calvete JJ. Integrated Venomics and Venom Gland Transcriptome Analysis of Juvenile and Adult Mexican Rattlesnakes Crotalus simus, C. tzabcan, and C. culminatus Revealed miRNA-modulated Ontogenetic Shifts. *J Proteome Res*. American Chemical Society; 2017; doi: 10.1021/acs.jproteome.7b00414.
126. Laxme RRS, Khochare S, Souza HF de, Ahuja B, Suranse V, Martin G, et al.. Beyond the 'big four': Venom profiling of the medically important yet neglected Indian snakes reveals disturbing antivenom deficiencies. *PLOS Neglected Tropical Diseases*. Public Library of Science; 2019; doi: 10.1371/journal.pntd.0007899.
127. Kimura M. Evolutionary Rate at the Molecular Level. *Nature*. 1968; doi: 10.1038/217624a0.
128. Wright S. Evolution in Mendelian Populations. *Genetics*. 16:97–1591931;
129. Margres MJ, Bigelow AT, Lemmon EM, Lemmon AR, Rokyta DR. Selection To Increase Expression, Not Sequence Diversity, Precedes Gene Family Origin and Expansion in Rattlesnake Venom. *Genetics*. 2017; doi: 10.1534/genetics.117.202655.
130. Oguiura N, Collares MA, Furtado MFD, Ferrarezzi H, Suzuki H. Intraspecific variation of the crotamine and crotasin genes in Crotalus durissus rattlesnakes. *Gene*. 2009; doi: 10.1016/j.gene.2009.05.015.
131. Charlesworth B. Effective population size and patterns of molecular evolution and variation. *Nat Rev Genet*. 2009; doi: 10.1038/nrg2526.
132. Ludington AJ, Sanders KL. Demographic analyses of marine and terrestrial snakes (Elapidae) using whole genome sequences. *Molecular Ecology*. 2021; doi: https://doi.org/10.1111/mec.15726.

133. Ochoa A, Broe M, Moriarty Lemmon E, Lemmon AR, Rokyta DR, Gibbs HL. Drift, selection and adaptive variation in small populations of a threatened rattlesnake. *Molecular Ecology*. 2020; doi: 10.1111/mec.15517.

Table 2. Whole-genome sequencing studies on snakes, published or in progress.

Scientific classification				Sequencing		Assembly				Annotation	Notes	
						Scaffold		Contig	Genome Size			
Superfamily	Family	Genus	Species	Sequencing platform	DoC	GC%	N50 Size (kb)	N50 Size (kb)	Genome Size (Gb)	Protein encoding genes identified	INSDC ID	Ref.
Colubroidea	Viperidae	*Bothrops*	*B. jararaca*	Illumina; PacBio; BAC-SeqSc	20 PL 150 IL	-	-	163.5	2.1	-	PRJNA691605	[27]
		Crotalus	*C. viridis*	Illumina; PacBio	100	36.6	139	15.74	1.3	-	PDHV00000000.1	[18,19]
			C. tigris	Illumina; PacBio	33 PL 190 IL	39.9/ 39.8	207,720	2,110	1.6	18, 240	VORL00000000	[26]
			C. pyrrhus	Illumina	40	38.5	5.1	4.1	1.1	−	JPMF00000000.1	[24]
			C. horridus	Illumina	135	34.3	23.8	5.8	1.5	−	LVCR00000000.1	[79]
		Protobothrops	*P. flavoviridis*	Illumina	96	38.2	467	3.8	1.4	20,540	BFFQ00000000.1	[13]
			P. mucrosquamatus	Illumina	86	40.6	424	22	1.6	20,122	BCNE00000000.2	[22]
		Sistrurus	*S. catenatus*	Illumina, PacBio	-		1,045,000	-	1.6	-	PRJNA750087	[29]
		Vipera	*V. berus*	Illumina	121	41.3	126.6	11.7	1.5	−	JTGP00000000.1	[23]
		Deinagkistrodon	*D. acutus*	Illumina	♂ 114 ♀ 238	-	2,120	22.42	1.4	21, 194	DQ343647.1	[88]
	Colubridae	*Pantherophis*	*P. guttatus*	Illumina	13	38.3	4.3	2.39	1.4	−	JTLQ00000000.1	[20]
		Thermophis	*T. baileyi*	Illumina	185	43.6	2,414	16.8	1.8	20,995	QLTV00000000	[21]
		Thamnophis	*T. sirtalis*	Illumina	72	41.8	516	10.45	1.4		LFLD00000000.1	[82]
			T. elegans	Illumina PacBio	62	41.1	100,851	4,620	1.6	18,900	PRJNA561996	
		Ophiophagus	*O. hannah*	Illumina	28	40.6	226	3.98	1.6		AZIM00000000.1	[16]

	Elapidae	*Pseudonaja*	*P. textilis*	–	73	40.1	14,685	50.44	1.6	19,358	ULFR00000000.1	[81]
		Notechis	*N. scutatus*	Illumina; PacBio	71	40.2	5,997	31.76	1.6	19,770	PRJEB27871	[26]
		Naja	*N. naja*	PacBio; Nanopore; Illumina	250	40.4	223,350	303.98	1.79	23,248	SOZL00000000.1	[25]
		Hydrophis	*H. curtus*	Illumina NovaSeq	120	37.2	1,346	183	1.62	21,863	PRJNA597425	[28]
Pythonoidea	Pythonidae	*Python*	*P. bivittatus*	Illumina; Roche 454	20	39.7	214	10.66	1.4	19,793	AEQU00000000.2	[7]
Booidea	Boidae	*Boa*	*B. constrictor*	Illumina; Roche 454; PacBio	125				1.6			[17]

GC% refers to the percentage of the Guanine (G) and Cytosine (C) bases in a genome, scaffold N50 is a measure of the assembly quality (see below), DoC is a measure of the depth of coverage (see below), and INSDC ID is the NCBI gene bank accession number of the respective genome. PB stands for PacBio and IL for Illumina.

Figure 1

Click here to access/download;Figure;Figure 1.png

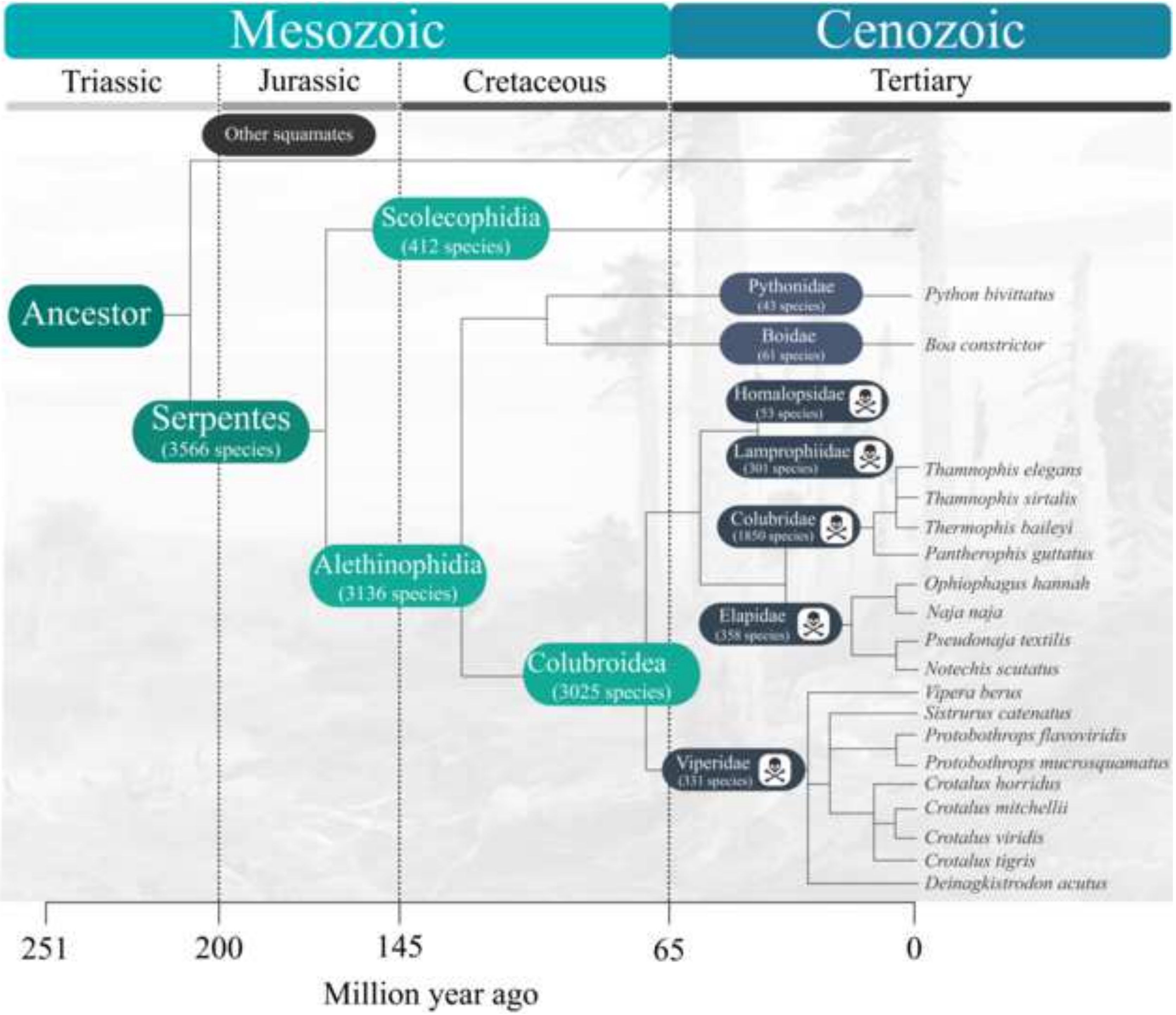

 Click here to access/download;Figure;Figure 2.png

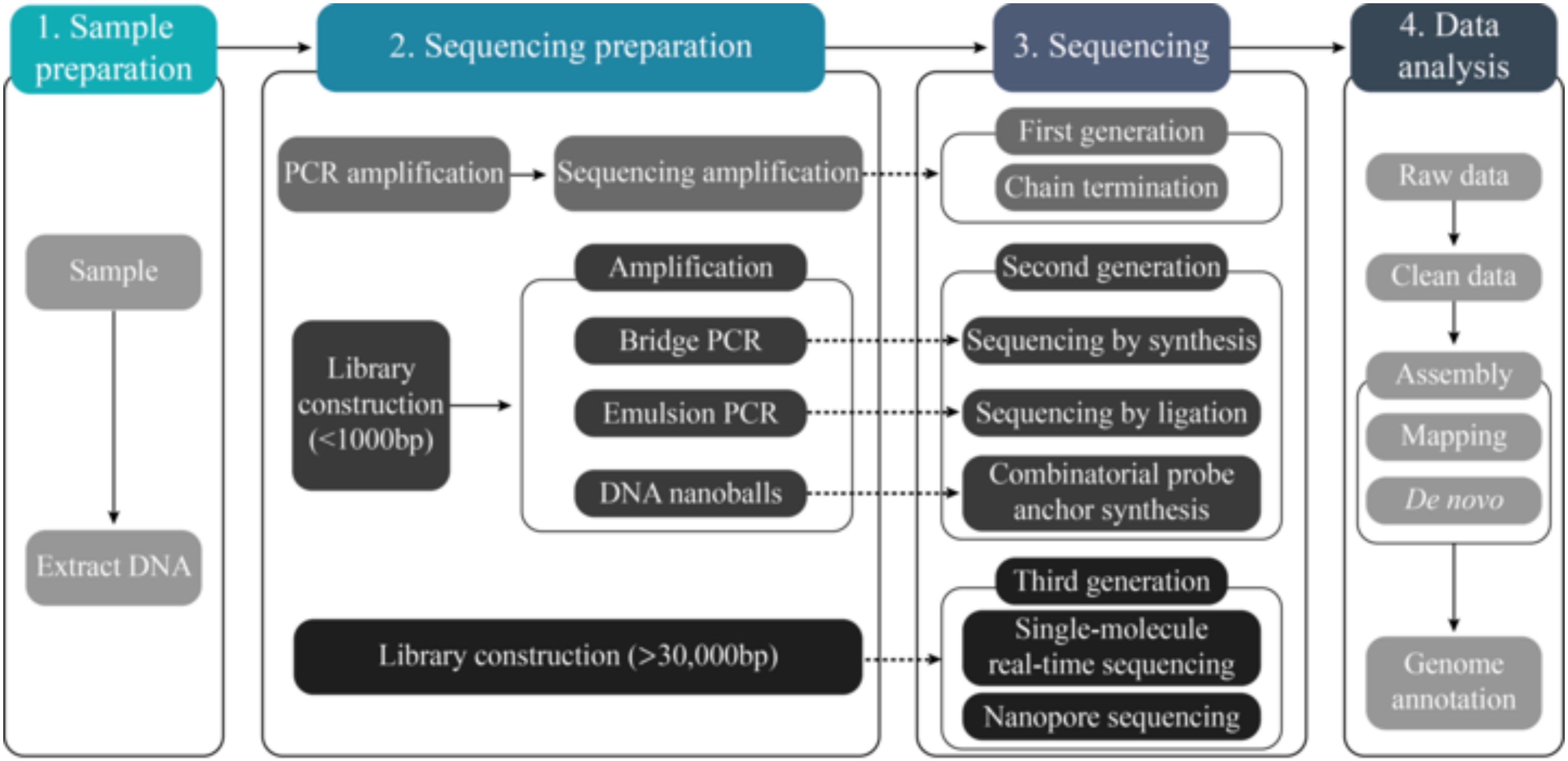

Figure 3

Click here to access/download;Figure;Figure 3.png

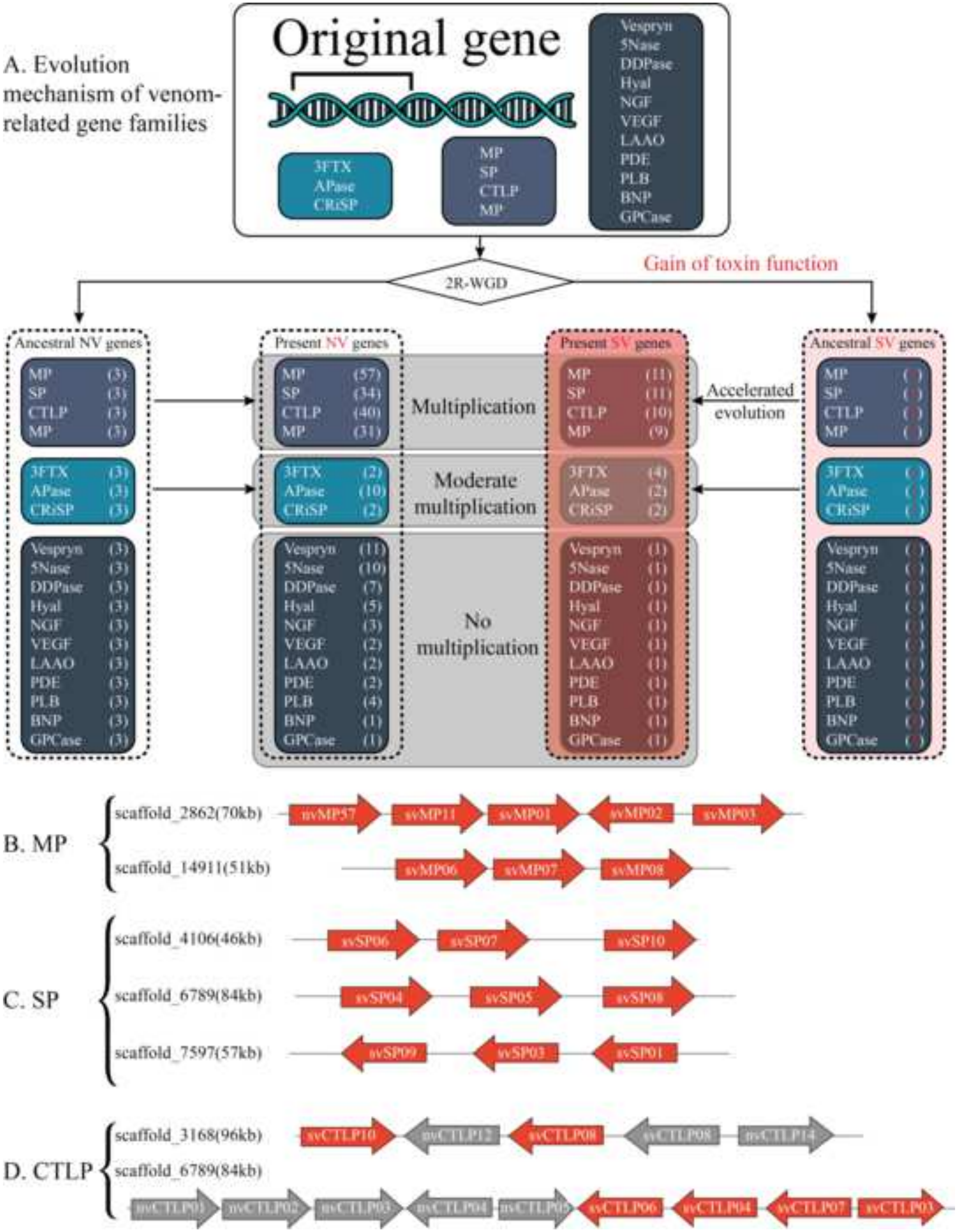

Figure 4

Click here to access/download;Figure;Figure 4.png

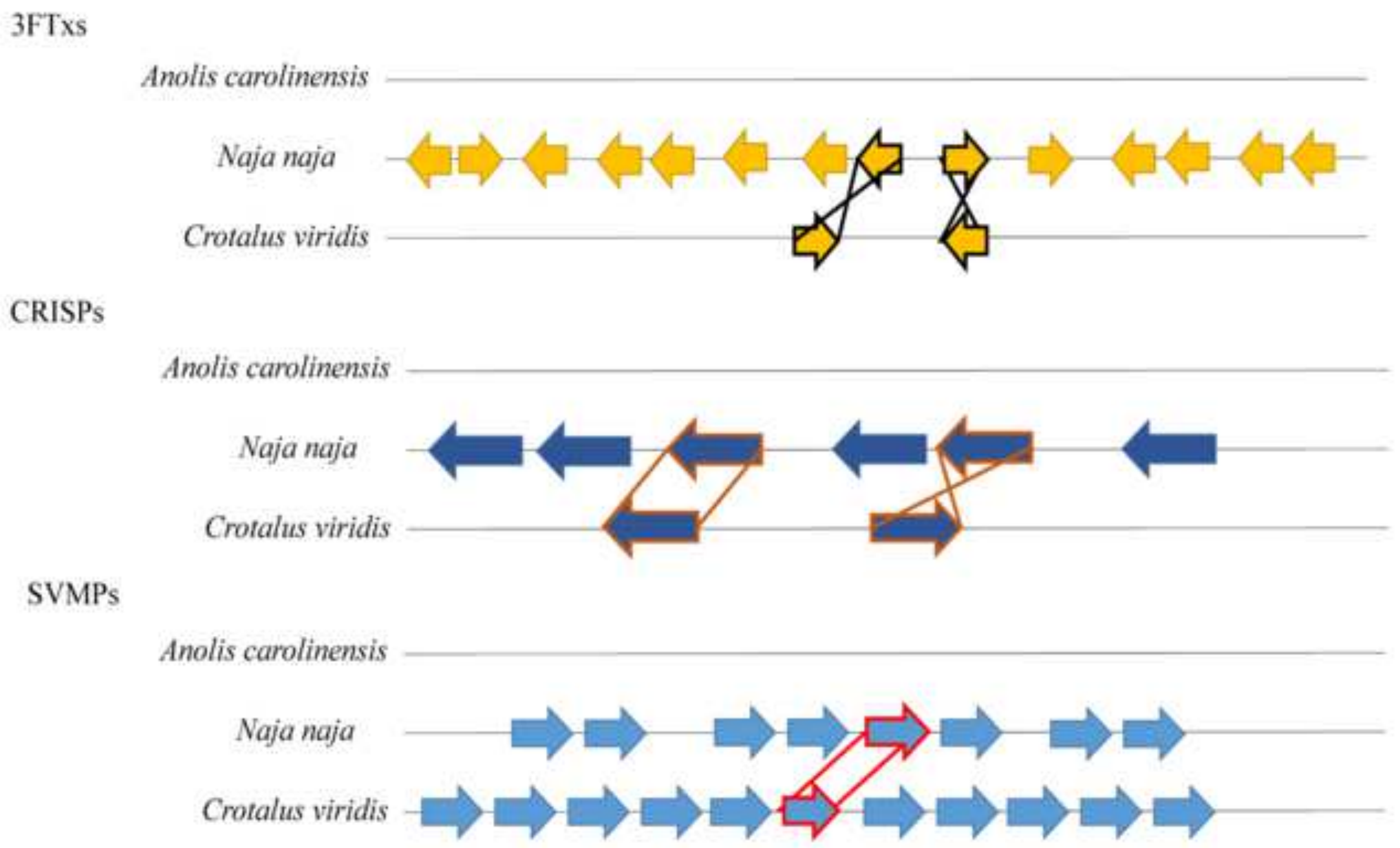

Supplement: giac024_GIGA-D-21-00410_Original_Submission [file giac024_giga-d-21-00410_original_submission.pdf]
