# Supplementary material for: The rise of genomics in snake venom research: recent advances and future perspectives
Source: Gigascience. 2022 Apr 1;11:giac024. doi: 10.1093/gigascience/giac024 (PMC8975721; doi:10.1093/gigascience/giac024)
Supplement: giac024_Response_to_Reviewer_Comments_Revision_1 [file giac024_response_to_reviewer_comments_revision_1.pdf]

Author's Response To Reviewer Comments

Close

We would like to thank the reviewers and the editor for a fast and very constructive review process. We have attempted to address the reviewer comments as good as possible, but please be critical about our responses/corrections, if you feel they or the manuscript could be further improved!

Reviewer #1: In this review, the authors provide an overview of recent applications of genomic analysis to study snake venom systems. This review covers an impressive span of topics related to genomics, snake venom, and the intersection of the two, and includes details from many relatively recent studies. There is minimal synthesis of the topics covered beyond echoing the major conclusions of the cited studies, and similar reviews have been published on this topic recently (Schield et al. 2021; Zancolli & Casewell, 2020).

I have several specific comments and requested edits that I feel should be addressed prior to the publication of this manuscript:
Response: References has been included to support the statement (lines 335-337).

Line 314-316: Include citations corresponding to the "uncertainty and controversy" about the origin of venom (for example, Hargreaves et al 2014). I do not think that it is accurate to conclude that one hypothesis "is generally accepted" on line 315. This could instead say that a prevailing hypothesis is that the core venom system evolved in the common ancestor of snakes and lizards, but that there are other competing hypotheses as well.
Response: We have modified the sentence to defined the origin of the venom system as “prevalent hypothesis” rather than “generally accepted and we have included the suggested reference.

Section 3.4 – I would suggest that the authors add a statement at the end of the first paragraph noting that relatively few methods have been used to study selection in venom genes, and have done so in a small number of species. Additional studies are needed before broad conclusions can be made about selection on venom genes. In fact, some studies have found evidence for other modes of selection acting on venom regions (i.e., balancing selection; Rautsaw et al 2019).
Response: We have included and additional paragraph at the end of the section (lines 618-623) and referenced as suggested.

Figure 1 - the caption says the data is from snakedatabase.org, however the citation refers to reptile-database.org. Please correct the caption to be consistent with the citation (reptile-databse.org).
Response: Corrected.

Table 1 - this table would be more informative if additional species were included. Specifically, only 2 of the 5 species in the table are venomous (Deinagkistrodon and Ophiophagus). As this review aims to be comprehensive, I think all currently available venomous snake data should be summarized here. Additions should include at least Naja naja (Suryamohan et al 2020), Crotalus viridis (Schield et al 2019), Crotalus tigris (Margres et al 2021), Thamnophis sirtalis (a rear-fanged venomous colubrid; Perry et al 2018), Protobothrops flavoviridis (Shibata et al 2018).
Response: We acknowledge the improvement upon implementation of the reviewer suggestion. The table has been updated with the suggested species and now, they are separared as non-venomous/venomous. A disclaimer has been included in the table description to highlight that the gene numbers for non-venomous species are homologue proteins found in other organs rather than in venom glands as listed for venomous species.

Tables 1 and 2 - indicate which species are and are not considered venomous.
Response: Table 1 has been reformatted to differentiate venomous and non-venomous snakes, and table2 include a new column with this information

Line 231 - cite the NCBI accession for the T. elegans assembly

Response: Reference included

Line 271 - typo, delete "0" from "0rattlesnake"
Response: Corrected.

Reviewer #2: Review: GIGA-D-21-00410, The rise of genomics in snake venom research: recent advances and future perspectives

The authors present a general review of the state of genomics of snakes and their venoms, with specific focus on what we stand to learn about venom function and evolution from individual snake genomes and subsequent comparative genomics. The authors' approach is very general, covering several components. In some places the authors' include specific details from the several published genome studies in snakes. These details are welcome, and in some places further clarification is needed. The paper will be a nice primer for someone starting to think about the many promises and complexities of genomics and venom genomics in particular, and highlights some of the things we stand to learn from additional genomes that are sure to come in the future. I suggest several places for revisions that, with major revision of both wording and associated figures, can lead to a clearer and more impactful manuscript. Sincerely, Matt Holding.

Major:

Line 55-58, and Figure 1: These lines and the topology of the tree in the figure are somewhat misleading. First, saying snakes "evolved from lizards" belies the complexity here. Snakes are actually a very successful lineage of lizards. This leads to the more important point…calling snakes and lizards "two groups" may make sense colloquially, but not phylogenetically. Several recent studies have shown that Serpentes is the sister group to Anguimorph lizards, and that these two are therefore more closely related to one another than to other lizards. As such, from a perspective of monophyly, lizards and snakes do not form "two groups". Moreover, the topology in Figure 1 is therefore incorrect, as it shows Serpentes sharing a common ancestor with presumably all "Other squamates", when in fact Serpentes + Anguimorph lizards share a common ancestor, and that ancestral lizard shared a common ancestor with "Other squamates"

https://bmcecolevol.biomedcentral.com/articles/10.1186/1471-2148-13-93
https://royalsocietypublishing.org/doi/full/10.1098/rsbl.2012.0703

Response: Thank you for the suggested references. The text has been updated (lines 55-59) and the references has been included.

Llines 221-222: Authors should expand here as a lead-in to the downstream discussions of studying venom genes with whole genomes. You mention "multi-gene families", but the real need for high quality contiguous genomes comes from tandemly array "gene islands", which is of course the case for all of the major venom gene families that you review in depth. It is therefore worth specific mention here and later that tandem duplication of similar sequence presents a particularly hard challenge for assembly software, and therefore long reads are needed for the best quality assessments of venom genes. Lines 388-393 contain the current mention of these gene islands. They are hard too assemble without PacBIO HiFi or Minion long reads, and this should be mentioned extensively in a paper like this. One.
Response: Good point, we agree with the Reviewer that long-read technology should be specifically mentioned here, as they are essential for the assembly of continuous genomes. We have therefore added two sentences in this paragraph that hopefully make the point clearer now, with a reference to Figure 2 where the long-read technology is also mentioned (lines 232-236).

Lines 346-347: A few lines to convince the reader that these differences aren't attributable to differences in the sequencing technologies (and therefore the ability to recover repetitive elements) would be appropriate here.
Response: We have included a paragraph exemplifying that the sequencing pproaches are not responsible for the registeres differences and references accordingly (lines 369-373)

Line 366-367: Where is the hypothesis coming from? There is no citation and it hasn't been previously mentioned. Why would microsatellites impact venom evolution? Are they enriched near venom genes?

Otherwise this pure speculation needs to be removed.
Response: We have removed the statement.

Lines 382 to 387: This is presented as if these are surprising instances of convergence. Rather, these are clearly homologous locations of these tandemly arrayed gene islands.
Response: We have modified the text clarified the poinr and included a statement abour the higher GC-content and faster recombination of macrocromosomes (lines 406-407 and 414-416).

Line 479-484: This is confusingly written. Which gene family(s) are you talking about? Name them rather than just saying "Toxin genes". The presentation of the idea in figure 3 doesn't help the confusion, but rather worsens it. MP, SP, CTL, etc., are large gene superfamilies, so of course they are all over the genome of vertebrates, and very few are venom. You need to undertake much clearer writing to get your point across and link the verbiage here to the figure, or remove this content about whole genome duplications completely and just stick to the better understood components of venom evolution. If you think there is clear evidence for an importance of these more ancient WGD events in venom gene evolution, then it certainly merits a place in the paper. But if so, this needs much more detail to differentiate the idea of more recent common ancestors of the venom progenitor genes from the broader gene superfamilies (most of which are housekeeping genes) to which these venom genes belong.
Response: We have detailed the toxin gene families studied in the P. flavoridis genome and modified the wording to point across figure 3.

Line 486-491: This is a major misrepresentation of these results. The scaffolds presented are tiny, on the order of only kilobases. Based on the more complete chromosome-scale assemblies done in Crotalus, it is clear that SVSP, SVMP, PLA2, and CTLP all occur in single gene clusters in pitvipers. You need to differentiate genomic scaffolds (a technical artifact of assembly quality) from gene clusters (a biological reality of genomic architecture). And then when doing so, it will always be best to default to the findings of those genomes that are done at chromosome scale, rather than in thousands of tiny scaffolds, until better genomes come out for the other species. See work from Sean Carroll's lab scaffolding individual gene familes, or the Crotalus viridis or C. tigris genomes.
Response: Thank you for pointing out. We acknowledge the misunderstanding in the terminology. We have modified the text to make it clear that we are referring to gene clusters rather than scaffolds (lines 498-503).

Minor:

Lines 117-120: Unclear why you would mention RADseq, as it is highly unlikely to hit a venom gene. Alternately, you could expand that it could help reveal potential population demographic trends that underly venom variation, as we did here:
https://academic.oup.com/biolinnean/article/132/2/297/6042638?login=true
Response: We thank the Reviewer for a good point here. We have chosen to still make a mention about the use of RAD-seq technology, to exemplify to the community another RRS technique that could be useful to study venom evolution. We have modified the sentence as suggested by the Reviewer by adding the Holding et al. study as a direct example of the use of RAD-seq for this purpose while acknowledging that it is less suitable for the study of venom genes (lines 119-124).

Line 297: This also implies greater overall gene density in T. sirtalis (genes/Mb) and potentially differences is repetitive element content. The authors should list out these possible causes and then review which are supported by the literature, rather than just mentioning average intron length differences, which amount to a relatively small overall amount of any genome. Tim
Response: We acknowledge that this paragraph needed clarification. We have modified the text to include the considerations about the gene density and included references to support the statements (lines 311-319).

Line 322: There is little to no evidence that "environmental conditions" impact venom gene expression. Clarify this statement. What does matter is population of origin, age of snake, and time since last expulsion of venom.
Response: We have clarified the point as suggested and included supporting references (lines 343-344).

Line 336: "fluid genome" is a very imprecise term. I think you want to say "high degree of evolvability in structural features of the genome".

Response: We thank the Reviewer for the suggestion. We have chosen to remove the term and change it for the suggestion made by the Reviewer (lines 357-358).

Line 468: "chromatin-wise" is unclear wording. Reword.
Response: We have modified the text to emphasize that highly expressed toxin genes are usually on regions where chromatin is available and the methylation allows for higher transcription of the genes, when compared with non toxin genes (lines 491-492).

Line 470: "metavenom network" is also not a generally known, well-defined term. Define or reword to be more specific.
Response: We have reworded the sentence and defined “metavenom network” in the text (line 495).

Line 479-484: This is confusingly written. Which specific gene family(s) are you talking about? Name them rather than just saying "Toxin genes".
Response: We have modified the text and included a description of the specific toxin families (lines 504-513)

Close
