# Supplementary material for: The rise of genomics in snake venom research: recent advances and future perspectives
Source: Gigascience. 2022 Apr 1;11:giac024. doi: 10.1093/gigascience/giac024 (PMC8975721; doi:10.1093/gigascience/giac024)
Supplement: giac024_Reviewer_1_Report_Original_Submission — Blair Perry -- 1/18/2022 Reviewed [file giac024_reviewer_1_report_original_submission.pdf]

Reviewer Report

Title: The rise of genomics in snake venom research: recent advances and future perspectives

Version: Original Submission **Date:** 1/18/2022

Reviewer name: Blair Perry

Reviewer Comments to Author:

In this review, the authors provide an overview of recent applications of genomic analysis to study snake venom systems. This review covers an impressive span of topics related to genomics, snake venom, and the intersection of the two, and includes details from many relatively recent studies. There is minimal synthesis of the topics covered beyond echoing the major conclusions of the cited studies, and similar reviews have been published on this topic recently (Schield et al. 2021; Zancolli & Casewell, 2020).
I have several specific comments and requested edits that I feel should be addressed prior to the publication of this manuscript:
Line 314-316: Include citations corresponding to the "uncertainty and controversy" about the origin of venom (for example, Hargreaves et al 2014). I do not think that it is accurate to conclude that one hypothesis "is generally accepted" on line 315. This could instead say that a prevailing hypothesis is that the core venom system evolved in the common ancestor of snakes and lizards, but that there are other competing hypotheses as well.
Section 3.4 - I would suggest that the authors add a statement at the end of the first paragraph noting that relatively few methods have been used to study selection in venom genes, and have done so in a small number of species. Additional studies are needed before broad conclusions can be made about selection on venom genes. In fact, some studies have found evidence for other modes of selection acting on venom regions (i.e., balancing selection; Rautsaw et al 2019).
Figure 1 - the caption says the data is from snakedatabase.org, however the citation refers to reptile-database.org. Please correct the caption to be consistent with the citation (reptile-databse.org).
Table 1 - this table would be more informative if additional species were included. Specifically, only 2 of the 5 species in the table are venomous (Deinagkistrodon and Ophiophagus). As this review aims to be comprehensive, I think all currently available venomous snake data should be summarized here. Additions should include at least Naja naja (Suryamohan et al 2020), Crotalus viridis (Schield et al 2019), Crotalus tigris (Margres et al 2021), Thamnophis sirtalis (a rear-fanged venomous colubrid; Perry et al 2018), Protobothrops flavoviridis (Shibata et al 2018).
Tables 1 and 2 - indicate which species are and are not considered venomous.
Line 231 - cite the NCBI accession for the T. elegans assembly
Line 271 - typo, delete "0" from "0rattlesnake"

Methods

Are the methods appropriate to the aims of the study, are they well described, and are necessary controls included? Choose an item.

Conclusions

Are the conclusions adequately supported by the data shown? Choose an item.

Reporting Standards

Does the manuscript adhere to the journal's guidelines on minimum standards of reporting? Choose an item.

Choose an item.

Statistics

Are you able to assess all statistics in the manuscript, including the appropriateness of statistical tests used? Choose an item.

Quality of Written English

Please indicate the quality of language in the manuscript: Choose an item.

Declaration of Competing Interests

Please complete a declaration of competing interests, considering the following questions:

- Have you in the past five years received reimbursements, fees, funding, or salary from an organisation that may in any way gain or lose financially from the publication of this manuscript, either now or in the future?
- Do you hold any stocks or shares in an organisation that may in any way gain or lose financially from the publication of this manuscript, either now or in the future?
- Do you hold or are you currently applying for any patents relating to the content of the manuscript?
- Have you received reimbursements, fees, funding, or salary from an organization that holds or has applied for patents relating to the content of the manuscript?
- Do you have any other financial competing interests?
- Do you have any non-financial competing interests in relation to this paper?

If you can answer no to all of the above, write 'I declare that I have no competing interests' below. If your reply is yes to any, please give details below.

I declare that I have no competing interests.

I agree to the open peer review policy of the journal. I understand that my name will be included on my report to the authors and, if the manuscript is accepted for publication, my named report including any attachments I upload will be posted on the website along with the authors' responses. I agree for my

report to be made available under an Open Access Creative Commons CC-BY license (http://creativecommons.org/licenses/by/4.0/). I understand that any comments which I do not wish to be included in my named report can be included as confidential comments to the editors, which will not be published.

Choose an item.

To further support our reviewers, we have joined with Publons, where you can gain additional credit to further highlight your hard work (see: https://publons.com/journal/530/gigascience). On publication of this paper, your review will be automatically added to Publons, you can then choose whether or not to claim your Publons credit. I understand this statement.

Yes Choose an item.
