# Supplementary material for: The rise of genomics in snake venom research: recent advances and future perspectives
Source: Gigascience. 2022 Apr 1;11:giac024. doi: 10.1093/gigascience/giac024 (PMC8975721; doi:10.1093/gigascience/giac024)
Supplement: giac024_Reviewer_2_Report_Original_Submission — Matthew Holding -- 1/18/2022 Reviewed [file giac024_reviewer_2_report_original_submission.pdf]

Reviewer Report

Title: The rise of genomics in snake venom research: recent advances and future perspectives

Version: Original Submission **Date:** 1/18/2022

Reviewer name: Matthew Holding

Reviewer Comments to Author:

Review: GIGA-D-21-00410, The rise of genomics in snake venom research: recent advances and future perspectives

The authors present a general review of the state of genomics of snakes and their venoms, with specific focus on what we stand to learn about venom function and evolution from individual snake genomes and subsequent comparative genomics. The authors' approach is very general, covering several components. In some places the authors' include specific details from the several published genome studies in snakes. These details are welcome, and in some places further clarification is needed. The paper will be a nice primer for someone starting to think about the many promises and complexities of genomics and venom genomics in particular, and highlights some of the things we stand to learn from additional genomes that are sure to come in the future. I suggest several places for revisions that, with major revision of both wording and associated figures, can lead to a clearer and more impactful manuscript. Sincerely, Matt Holding.

Major:

Line 55-58, and Figure 1: These lines and the topology of the tree in the figure are somewhat misleading. First, saying snakes "evolved from lizards" belies the complexity here. Snakes are actually a very successful lineage of lizards. This leads to the more important point…calling snakes and lizards "two groups" may make sense colloquially, but not phylogenetically. Several recent studies have shown that Serpentes is the sister group to Anguimorph lizards, and that these two are therefore more closely related to one another than to other lizards. As such, from a perspective of monophyly, lizards and snakes do not form "two groups". Moreover, the topology in Figure 1 is therefore incorrect, as it shows Serpentes sharing a common ancestor with presumably all "Other squamates", when in fact Serpentes + Anguimorph lizards share a common ancestor, and that ancestral lizard shared a common ancestor with "Other squamates"

https://bmcecolevol.biomedcentral.com/articles/10.1186/1471-2148-13-93

https://royalsocietypublishing.org/doi/full/10.1098/rsbl.2012.0703

Llines 221-222: Authors should expand here as a lead-in to the downstream discussions of studying venom genes with whole genomes. You mention "multi-gene families", but the real need for high quality contiguous genomes comes from tandemly array "gene islands", which is of course the case for all of the major venom gene families that you review in depth. It is therefore worth specific mention here and later that tandem duplication of similar sequence presents a particularly hard challenge for assembly software, and therefore long reads are needed for the best quality assessments of venom genes. Lines 388-393 contain the current mention of these gene islands. They are hard too assemble without PacBIO HiFi or Minion long reads, and this should be mentioned extensively in a paper like this. One.

Lines 346-347: A few lines to convince the reader that these differences aren't attributable to differences in the sequencing technologies (and therefore the ability to recover repetitive elements) would be appropriate here.
Line 366-367: Where is the hypothesis coming from? There is no citation and it hasn't been previously mentioned. Why would microsatellites impact venom evolution? Are they enriched near venom genes? Otherwise this pure speculation needs to be removed.
Lines 382 to 387: This is presented as if these are surprising instances of convergence. Rather, these are clearly homologous locations of these tandemly arrayed gene islands.
Line 479-484: This is confusingly written. Which gene family(s) are you talking about? Name them rather than just saying "Toxin genes". The presentation of the idea in figure 3 doesn't help the confusion, but rather worsens it. MP, SP, CTL, etc., are large gene superfamilies, so of course they are all over the genome of vertebrates, and very few are venom. You need to undertake much clearer writing to get your point across and link the verbiage here to the figure, or remove this content about whole genome duplications completely and just stick to the better understood components of venom evolution. If you think there is clear evidence for an importance of these more ancient WGD events in venom gene evolution, then it certainly merits a place in the paper. But if so, this needs much more detail to differentiate the idea of more recent common ancestors of the venom progenitor genes from the broader gene superfamilies (most of which are housekeeping genes) to which these venom genes belong.
Line 486-491: This is a major misrepresentation of these results. The scaffolds presented are tiny, on the order of only kilobases. Based on the more complete chromosome-scale assemblies done in Crotalus, it is clear that SVSP, SVMP, PLA2, and CTLP all occur in single gene clusters in pitvipers. You need to differentiate genomic scaffolds (a technical artifact of assembly quality) from gene clusters (a biological reality of genomic architecture). And then when doing so, it will always be best to default to the findings of those genomes that are done at chromosome scale, rather than in thousands of tiny scaffolds, until better genomes come out for the other species. See work from Sean Carroll's lab scaffolding individual gene familes, or the Crotalus viridis or C. tigris genomes.
Minor:
Lines 117-120: Unclear why you would mention RADseq, as it is highly unlikely to hit a venom gene. Alternately, you could expand that it could help reveal potential population demographic trends that underly venom variation, as we did here:
https://academic.oup.com/biolinnean/article/132/2/297/6042638?login=true
Line 297: This also implies greater overall gene density in T. sirtalis (genes/Mb) and potentially differences is repetitive element content. The authors should list out these possible causes and then review which are supported by the literature, rather than just mentioning average intron length differences, which amount to a relatively small overall amount of any genome.
Line 322: There is little to no evidence that "environmental conditions" impact venom gene expression. Clarify this statement. What does matter is population of origin, age of snake, and time since last expulsion of venom.
Line 336: "fluid genome" is a very imprecise term. I think you want to say "high degree of evolvability in structural features of the genome".
Line 468: "chromatin-wise" is unclear wording. Reword.

Line 470: "metavenom network" is also not a generally known, well-defined term. Define or reword to be more specific.
Line 479-484: This is confusingly written. Which specific gene family(s) are you talking about? Name them rather than just saying "Toxin genes".

Methods

Are the methods appropriate to the aims of the study, are they well described, and are necessary controls included? Choose an item.

Conclusions

Are the conclusions adequately supported by the data shown? Choose an item.

Reporting Standards

Does the manuscript adhere to the journal's guidelines on minimum standards of reporting? Choose an item.

Choose an item.

Statistics

Are you able to assess all statistics in the manuscript, including the appropriateness of statistical tests used? Choose an item.

Quality of Written English

Please indicate the quality of language in the manuscript: Choose an item.

Declaration of Competing Interests

Please complete a declaration of competing interests, considering the following questions:

- Have you in the past five years received reimbursements, fees, funding, or salary from an organisation that may in any way gain or lose financially from the publication of this manuscript, either now or in the future?
- Do you hold any stocks or shares in an organisation that may in any way gain or lose financially from the publication of this manuscript, either now or in the future?
- Do you hold or are you currently applying for any patents relating to the content of the manuscript?
- Have you received reimbursements, fees, funding, or salary from an organization that holds or has applied for patents relating to the content of the manuscript?
- Do you have any other financial competing interests?

- Do you have any non-financial competing interests in relation to this paper?

If you can answer no to all of the above, write 'I declare that I have no competing interests' below. If your reply is yes to any, please give details below.

I declare that I have no competing interests

I agree to the open peer review policy of the journal. I understand that my name will be included on my report to the authors and, if the manuscript is accepted for publication, my named report including any attachments I upload will be posted on the website along with the authors' responses. I agree for my report to be made available under an Open Access Creative Commons CC-BY license (http://creativecommons.org/licenses/by/4.0/). I understand that any comments which I do not wish to be included in my named report can be included as confidential comments to the editors, which will not be published.

Choose an item.

To further support our reviewers, we have joined with Publons, where you can gain additional credit to further highlight your hard work (see: https://publons.com/journal/530/gigascience). On publication of this paper, your review will be automatically added to Publons, you can then choose whether or not to claim your Publons credit. I understand this statement.

Yes Choose an item.
